# Mechanical codes of chemical-scale specificity in DNA motifs†

**DOI:** 10.1039/d3sc01671d

**Published:** 2023-08-29

**Authors:** Yi-Tsao Chen, Haw Yang, Jhih-Wei Chu

**Affiliations:** a Institute of Bioinformatics and Systems Biology, National Yang Ming Chiao Tung University Hsinchu 30010 Taiwan Republic of China jwchu@nctu.edu.tw; b Department of Chemistry, Princeton University Princeton NJ 08544 USA; c Department of Biological Science and Technology, Institute of Molecular Medicine and Bioengineering, Center for Intelligent Drug Systems and Smart Bio-devices (IDS^2^B), National Yang Ming Chiao Tung University Hsinchu 30010 Taiwan Republic of China

## Abstract

In gene transcription, certain sequences of double-stranded (ds)DNA play a vital role in nucleosome positioning and expression initiation. That dsDNA is deformed to various extents in these processes leads us to ask: Could the genomic DNA also have sequence specificity in its chemical-scale mechanical properties? We approach this question using statistical machine learning to determine the rigidity between DNA chemical moieties. What emerges for the polyA, polyG, TpA, and CpG sequences studied here is a unique trigram that contains the quantitative mechanical strengths between bases and along the backbone. In a way, such a sequence-dependent trigram could be viewed as a DNA mechanical code. Interestingly, we discover a compensatory competition between the axial base-stacking interaction and the transverse base-pairing interaction, and such a reciprocal relationship constitutes the most discriminating feature of the mechanical code. Our results also provide chemical-scale understanding for experimental observables. For example, the long polyA persistence length is shown to have strong base stacking while its complement (polyA^c^) exhibits high backbone rigidity. The mechanical code concept enables a direct reading of the physical interactions encoded in the sequence which, with further development, is expected to shed new light on DNA allostery and DNA-binding drugs.

## Abstract 2

Could genomic DNA have sequence specificity in its chemical-scale mechanical properties? We approach this question using statistical machine learning to determine the rigidity between DNA chemical moieties.

## Graphical abstract

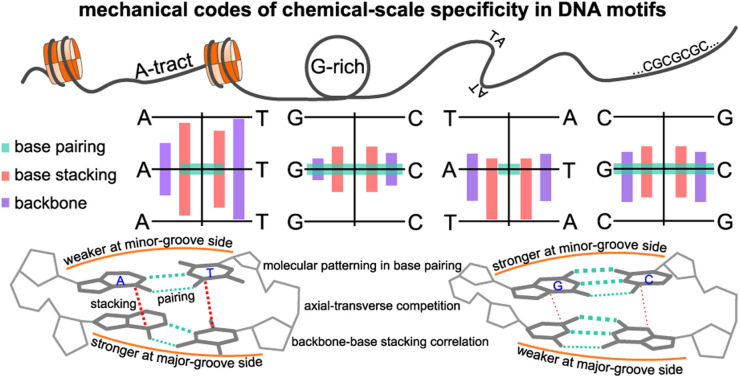

## 1 Introduction

The genome contains cues for regulating gene expression, and the sequence-dependent stiffness of DNA^1^ has been recognized as an essential property mediating such actions as nucleosome positioning,^2–4^ initiation control,^5–7^ and expression modulation,^8–10^ to name a few. Gene-regulation signals usually consist of a short stretch of double-stranded DNA (dsDNA) of defined pattern and deformation of such DNA segments was shown to be essential in these processes.^11–14^ Down to the chemical moiety level, various structural studies have reported complex patterns of DNA sequence dependence in base pairing geometries, ribose puckering, and backbone conformations that affect the manner by which regulatory proteins bind to DNA.^15–18^ These observations suggest that there may be a fundamental connection between the DNA sequence patterning and the larger-scale mechanical response;^1^ yet, how these are related to chemical-scale stiffness remains elusive to articulate. Knowledge of the connections linking chemical moieties to DNA mechanical response could facilitate new experimental designs and improve interpretation of functional genomics, provide new insights for the machinery in DNA-interacting proteins,^19^ and accelerate the development of DNA-binding drugs,^20,21^ for example.

To resolve such connection that crosses several time- and length-scales, here we use the recently developed structure-mechanics statistical learning framework^22^ and graph-theory analysis^23^ to quantify dsDNA rigidity within and between chemical moieties. It is found that each of the regulatory DNA sequences studied exhibits a distinct base-to-backbone chemomechanical linkage that can be likened to rigidity fingerprints. They serve as a unique and quantifiable dsDNA mechanical coupling presentation, and provide an intuitive understanding for experimental observations. This finding thus suggests an interesting new way of appreciating how local genetic information on the chemical moiety level is transmitted to influencing the large-scale biological function: mechanistically, sequence-specific information propagates not only by means of cognizant DNA-binding proteins but also throughout the DNA chain by physical-force based mechanical coupling. As each rigidity fingerprint is unique and context sensitive, in a sense, the rigidity fingerprint could be also viewed as a mechanical code.

**Fig. 1:**
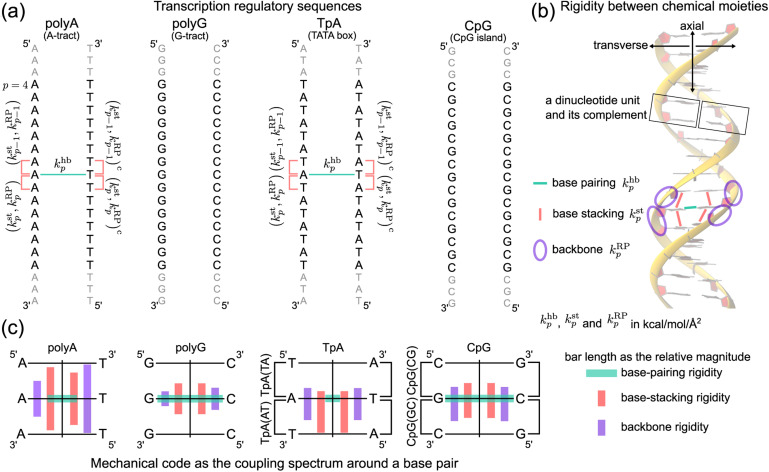
Nucleic acid mechanics in transcription regulation. (a) The 21-bp dsDNA studied in this work includes polyA, polyG, TpA, and CpG. At base *p*, the base-pairing rigidity due to hydrogen bonding (hb) is *k*^hb^_*p*_. Next to *k*^hb^_*p*_ in the reference strand is (*k*^st^_*p*_,*k*^RP^_*p*_), the rigidity of base stacking (st) and ribose-phosphate backbone (RP) toward the 3′-end, and (*k*^st^_*p*−1_,*k*^RP^_*p*−1_) toward the 5′-end. Similarly, the complementary strand has (*k*^st^_*p*_,*k*^RP^_*p*_)^c^ and (*k*^st^_*p*−1_,*k*^RP^_*p*−1_)^c^. The (*k*^st^_*p*−1_,*k*^RP^_*p*−1_)/(*k*^st^_*p*_,*k*^RP^_*p*_) − *k*^hb^_*p*_ − (*k*^st^_*p*−1_,*k*^RP^_*p*−1_)^c^/(*k*^st^_*p*_,*k*^RP^_*p*_)^c^ mechanical code is shown in polyA and TpA for illustration. (b) The inter-moiety rigidity of base pairing, base stacking, and backbone, a trigram, is statistically learned from all-atom MD data. (c) The mechanical code reduced to a single element around a base pair by virtue of symmetry in the transcription regulatory sequences studied here. The bar length indicates the relative magnitude of base-pairing (green), base-stacking (red), and backbone (purple) rigidity, and the mean rigidity *k*^hb^, *k*^st^, and *k*^RP^ as the average of *k*^hb^_*p*_, *k*^st^_*p*_, and *k*^RP^_*p*_ in each sequence are used to set the bar lengths. Notice that the mechanical code represented as the coupling spectrum around a base pair has symmetry with respect to the middle horizontal line in homopolymeric sequences and to the vertical line in ambigram systems.

In this first study on the mechanical code, we focus on four hallmark sequence motifs in transcription regulation (Fig. 1a): polyA, polyG, TpA, and CpG. The homopolymeric polyA (5–20 base pair (bp) A-tract^24–27^ of poly(dA:dT)) has unusual behaviors and plays important roles in nucleosome positioning,^28–32^ whereas polyG serves as a model for the G-rich repressor segment of a promoter.^33,34^ TpA is part of the TATA box for the initiation control of transcription and has axially alternating repeats of ambigram symmetry.^35–37^ The CpG sequence is involved in mammal gene expression and in epigenetic regulation.^38–40^

Our strategy can be understood as follows. To ensure that the chemical identities are expressively included, all-atom MD simulations of 21-bp polyA, polyG, TpA, and CpG sequences are performed in explicit water for 5 μs production runs. The atomistic MD data are used to evaluate the computational mechanical properties for quantitative comparison with experiments as detailed in the ESI.† The same MD data are also coarse-grained to mesoscale by statistical learning to compute the elastic parameters in the heavy-atom elastic network model (haENM) of dsDNA. This approach has been established to have specific elastic constants up to the nearest-neighbor moieties while the harmonic potentials between heavy atoms of a greater separation would have zero or negligible values through the statistical learning.^22^ Prediction of dsDNA thermal stability from sequence was also based on the parameters between the nearest neighbors.^41^ As such, analysis at the heavy-atom level allows quantification of rigidity between chemical moieties.

Our determination of haENM spring constants achieves self-consistency by going beyond a simple inversion of the covariance matrix,^42^ and is used to inform the holistic chemical-scale rigidity for mechanistic understanding of sequence specificity. On the other hand, conventional methods mostly focused on the rigid planes of bases and base pairs by their helical coordinates.^43–45^ Mechanical properties extracted using the collective variables defined that way, however, tend to scramble the chemically informative inter-molecular interactions. The chemical mechanism for such property as high twist flexibility or negative tilt–shift correlation is thus difficult to deduce and articulate. Alternatively, structure-mechanics statistical learning with haENM offers a way to resolve the inter-atomic couplings in DNA dynamics. For example, the sequence-dependent choreography of backbone and base movements was noticed,^18^ and it would be valuable to be able to trace the molecular origin. Furthermore, the interplay of the base pairing interaction with the base stacking interaction in giving rise to sequence specificity is mostly elusive.

**Fig. 2:**
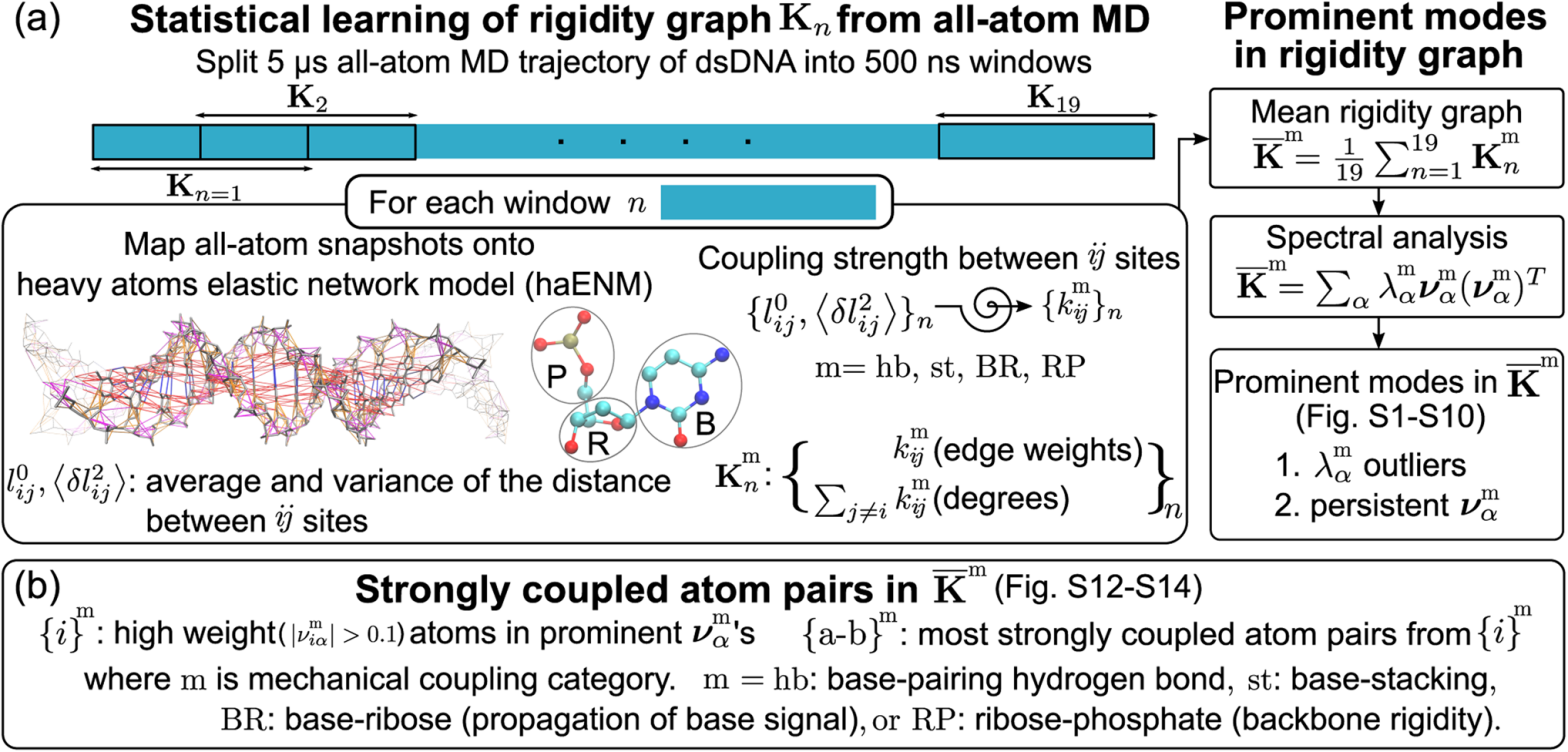
Quantification of inter-moiety rigidity in nucleic acids by structure-mechanics statistical learning with graph-theory analysis. (a) Flowchart of computing the haENM (heavy-atom elastic network model) spring constants from the production run all-atom MD trajectory and the assembly of rigidity graphs. The superscript m in *k*^m^_*ij*_ denotes the inter-moiety rigidity that it belongs to, m = hb, st, BR, or RP. The rigidity graph of the *n*th window, **K**^m^_*n*_, is constructed with the list of spring constants, {*k*^m^_*ij*_}_*n*_, and the mean rigidity graph **K̄**^m^ is the average of all **K**^m^_*n*_'s. Spectral analysis of **K̄**^m^ and all **K**^m^_*n*_'s is then conducted to identify the statistically prominent modes of the mean graph. (b) Spotting the list of most strongly coupled atom pairs {a–b}^m^ from **K̄**^m^. The other details are in the Materials and methods section.

We follow the previously established compartmentalization scheme to dissect molecular rigidity in chemical terms.^22^ These intertwining haENM springs could be visually informative when superimposed onto the dsDNA 3D structure (Fig. 2); however, they are still local to the mesoscale. This is where the techniques and concepts developed in the graph theory community come in. The graph-theory analysis detailed in the ESI† is used to quantitatively winnow the insignificant and at the same time concatenate the strongly coupled, regardless of the physical scale of the connections on which it operates—a cross-scale analysis. More specifically for this application, we generalize the graph-theory analysis framework initially developed for proteins^23^ and apply it to analyze the strengths of base-pairing hydrogen bonding (hb), base stacking (st), and ribose-phosphate backbone (RP) for the transcription regulatory sequences. What emerges is coupling pathways that thread through all scales and that are composed mostly of hb, st, and RP interactions (see Fig. 1b for an illustration of the various terms used in constructing the mechanical code). The rigidity is in the unit of kcal mol^−1^ Å^−2^, and the specific values of the hb, st, and RP categories form a trigram and constitute the mechanical code.

For each of the regulatory sequence motifs studied here, by virtue of symmetry, the mechanical code can be further reduced to a single element as the mean rigidity between chemical moieties around a base pair (Fig. 1c). Notice that the dinucleotide units in polyA and in its complement polyA^c^ (*cf.*Fig. 1b) are composed of different bases. TpA, on the other hand, has axially alternating TpA(AT) and TpA(TA) dinucleotide sequences of ambigram symmetry (the same nomenclature is applied to the dsDNA of G–C pairing). Therefore, the mechanical codes shown as the coupling spectra around a base pair in Fig. 1c exhibit symmetry with respect to the base-pairing plane (middle horizontal line) for homopolymeric sequences but along the complementarity interface (vertical line) in ambigram systems. These variations provide a visual example for context-sensitive signals of inter-moiety coupling as well an intuitive understanding of the relative mechanical strengths of these sequences. For example, the strong axial coupling in polyA indicates low bendability, while the very weak base-stacking and backbone rigidity at TpA(TA) suggest a higher chance of having kinks.

## 2 Materials and methods

The haENM spring constants are statistically learned from the 5 μs production run of all-atom MD trajectory. On this time-scale, dsDNA structural fluctuations were shown to exhibit convergent values,^46^ from which our results indicate that robust mechanical signals can be extracted with statistical significance. Since a non-hydrogen atom could be either in the phosphodiester bond (P), ribose (R), or base (B), a spring *k*^m^_*ij*_ is categorized as m = hb (base pairing), st (base stacking), BR (base–ribose linkage), or RP (ribose–phosphate backbone), Fig. 2a. Other harmonic potentials are mostly intra-moiety interactions that have very high spring constants and insignificant sequence dependence. Therefore, we focus on the hb, st, BR, and RP categories, and the rigidity graph of each is the heavy-atom nodes with their elastic parameters as edge weights for capturing the mechanical signals during dynamics. In the following, the other details of our graph-theory analysis for structure-mechanics statistical learning are discussed.

### 2.1 All-atom MD simulations

Nucleic Acid Builder^47^ is used to construct the B-form all-atom model of 21 bases for transcription regulatory sequences. Each system is solvated in a dodecahedron box of explicit water with at least 10 Å between any nucleic acid atom and box edges. K^+^ and Cl^−^ ions are added for charge neutrality and 0.15 M ionic strength. The AMBER BSC1 force field^48^ is employed to compute the potential energy and the GROMACS software^49^ is used for MD simulations. The cut-off radius for van der Waals interactions and real-space particle-mesh Ewald terms of electrostatics^50^ is 12 Å with a switching function effective at 10 Å. During the all-atom MD simulations, all bond lengths involving hydrogen are constrained *via* LINCS.^51^ After initial minimization and 12 ns equilibration period, the production run of 5 μs is conducted at constant temperature (310 K) and pressure (1.013 bar) *via* the Langevin thermostat and the Parrinello–Rahman barostat.^52^ A snapshot is saved every 100 ps for learning haENM spring constants and other properties.

### 2.2 Calculation of persistence length from dsDNA dynamics

Persistence length is defined by viewing dsDNA as a linear curve with tangent vectors along the contour length. The helical axis of each atomistic configuration sampled in the MD simulations of dsDNA is computed by curves+^53^ for determining the contour length and the tangent vectors. The Fourier mode amplitudes of the bending deformation are then evaluated, and their variances in the trajectory are used to calculate persistence length *L*_p_.^54^ Other details are reported in the ESI.†

For the 21-bp transcription regulatory sequences, the persistence length calculated from the 5 μs all-atom MD trajectory is polyA *L*_p_ = 71.8 ± 3.7 nm, polyG *L*_p_ = 49.1 ± 2.5 nm, TpA *L*_p_ = 53.3 ± 5.7 nm, and CpG *L*_p_ = 66.5 ± 2.6 nm. PolyA and CpG appear to be stiffer with a longer *L*_p_ whereas polyG and TpA are more flexible. TpA and polyG have *L*_p_ values around the 51 nm result based on a generic sequence measured with atomic force microscopy (AFM).^12^ The TpA *L*_p_ being slightly longer than that of polyG was also observed in cyclization experiments.^55^ PolyA having ultra high bending rigidity is in agreement with gel electrophoresis studies,^24,25^ and the *L*_p_ value in our calculation (71.8 nm) is quantitatively close to the result of a knowledge-based model.^56^ The calculated CpG *L*_p_ (66.5 nm) is also similar to the result based on AFM.^57^ This value is only slightly lower than the polyA *L*_p_, reflecting the role of CpG sequences in enhancing the bending rigidity of long dsDNA sequences.^58^

### 2.3 Construction of rigidity graphs from dsDNA dynamics

The mechanical coupling network of each DNA sequence is represented by haENM with the *k*_*ij*_'s as edge weights, Fig. 2a. Here, *i* and *j* are the number indices of atoms. All heavy-atom pairs with the averaged distances within the 4.7 Å cutoff^22^ are connected by a spring in the haENM. The difficulty of modeling nucleic acids by ENM^59^ is tackled here by structure-mechanics statistical learning. ENM is widely used in modeling protein systems,^60,61^ and our approach can also be adopted to understand the very complicated structural dynamics.^62,63^ From the all-atom trajectory data, the calculated variances for this list of inter-atomic distances, 〈δ*l*_*ij*_^2^〉_AA_'s, are used to statistically learn the *k*_*ij*_ values by the self-consistent iteration of *k*^(*n*+1)^_*ij*_ = *k*^(*n*)^_*ij*_ + *η*(1/〈δ*l*_*ij*_^2^〉^(*n*)^_NMA_ − 1/〈δ*l*_*ij*_^2^〉_AA_); 〈δ*l*_*ij*_^2^〉^(*n*)^_NMA_ is the variance predicted by normal mode analysis (NMA) of haENM, (*n*) is the iterative step, and *η* is a numerical learning factor which is kept constant. Since the springs between dsDNA heavy atoms are interconnected, the self-consistent iteration is to tackle the coupled statistics of different mechanical interactions.

With haENM giving a harmonic approximation, force-field anharmonicity and long-term dynamics would cause the *k*_*ij*_ values to vary. As an effective way for examining edge weight variation, the 5 μs all-atom MD trajectory is split into overlapping 500 ns windows,^23^ and the set of haENM parameters of each window *n*, {*k*_*ij*_}_*n*_, is calculated by the aforementioned structure-mechanics statistical learning as indicated in Fig. 2a. In a graph-theory representation of the *n*-th haENM, {*k*_*ij*_}_*n*_ is the off-diagonals of the square matrix **K**_*n*_ with the dimension of non-hydrogen atom sites, and the diagonal degrees of which are the sum over off-diagonals, Fig. 2a. Since each spring is categorized as m = hb, st, BR, or RP, {*k*^m^_*ij*_}_*n*_ and **K**^m^_*n*_ are the set of elastic parameters and rigidity graph, respectively, of category m. Other details are reported in the ESI.†

### 2.4 Identification of prominent patterns in rigidity graphs—the strongly coupled atom pairs

For each m = hb, st, BR, or RP, spectral decomposition of the mean rigidity graph averaged over **K**^m^_*n*_'s gives 
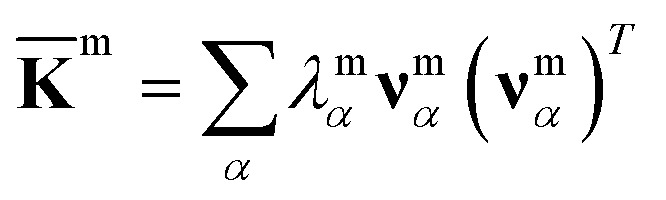
 that defines the mean-modes as **ν**^m^_*α*_ eigenvectors and *λ*^m^_*α*_ eigenvalues, Fig. 2a. For **ν**^m^_*α*_, the mean-mode content in each window is calculated as *r*^m^_*nα*_ = max_*β*_|**ν**^m^_*nβ*_·**ν**^m^_*α*_| with the **K**^m^_*n*_ eigenvectors of the window. The averaged mean-mode content across the production run, 〈*r*^m^_*α*_〉, is the persistence metric of mean-mode *α* in the mechanical compartment.^23^ Statistical outliers of the *λ*^m^_*α*_ distribution that also have high mean-mode contents (〈*r*^m^_*α*_〉 > 0.8) are then identified as the prominent modes of the mean rigidity graph, Fig. S1–S10.† Next, the set of high weight atoms in the prominent modes, {*i*}^m^, is used to identify the list of most strongly coupled atom pairs, {a–b}^m^, Fig. 2b. Spring constants of atom pairs in the {a–b}^m^ list are statistical outliers that represent the prominent couplings in **K̄**^m^.

Here, a and b are the names of the strongly coupled atoms (mechanical hotspots). For example, the {a–b}^hb^ lists of mechanical hotspots in the base pairing of polyA and polyG are expected to be related to the hydrogen bonds and are indeed identified to be {C2–O2, N1–N3, N6–O4} and {N2–O2, N1–N3, O6–N4}, which are consistent with the donor–acceptor notion in base pairing and serve as a validation that the above scheme of identifying prominent patterns in the rigidity graph can indeed capture the salient features of nucleic acid dynamics. The mechanical hotspots of the other inter-moiety rigidity of m = st, BR, or RP, though, are very difficult to expect *a priori* and can provide unprecedented insights as discussed later. For the inter-moiety rigidity in different sequences, the detailed statistics for identifying {a–b}^m^ are described in the ESI and the results are shown in Fig. S11–S14.†

### 2.5 Quantification of the rigidity between chemical moieties

**Fig. 3:**
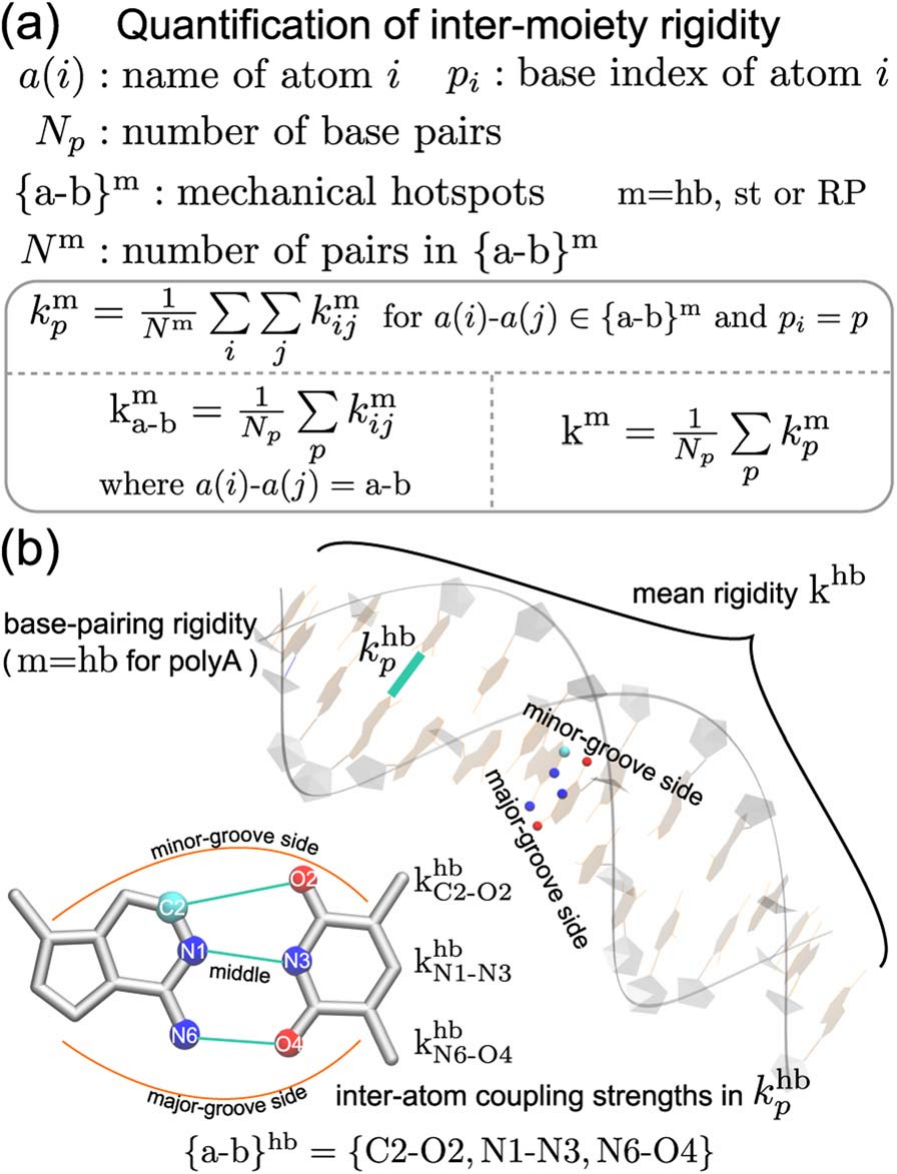
The inter-moiety rigidity of m = hb, st, or RP is calculated from haENM spring constants ({*k*^m^_*ij*_}) that are determined by structure-mechanics statistical learning with an all-atom MD trajectory. The {a-b}^m^ list of strongly coupled atom pairs (mechanical hotspots) is identified by the graph-theory analysis of {*k*^m^_*ij*_}, ESI.† (a) Mathematical definition of inter-moiety rigidity. The rigidity of inter-moiety m at base *p* is *k*^m^_*p*_. For an atom pair in the {a–b}^m^ list, *k*^m^_a–b_ is the averaged strength over all bases. Averaging the *k*^m^_*p*_ values of all bases gives *k*^m^, the mean inter-moiety rigidity. (b) Schematic representation of *k*^m^_*p*_, *k*^m^_a–b_, and *k*^m^ using m = hb in polyA as an example.

Compartmentalization of the haENM springs (*k*^m^_*ij*_'s) into m = hb, st, BR, or RP provides a way to quantify the rigidity between base, ribose, and backbone moieties. As listed in Fig. 3a, the inter-moiety rigidity at base *p*, *k*^m^_*p*_, is computed from the *k*^m^_*ij*_ values in which the *i* and *j* atoms are in either of the two interacting moieties. To focus on the statistically prominent restraints, the *k*^m^_*ij*_ values of atom pairs in the {a–b}^m^ list of very strongly coupled mechanical hotspots are averaged to determine *k*^m^_*p*_. For instance, since the {a–b}^hb^ of polyA is {C2–O2, N1–N3, N6–O4} (Fig. 3b), *k*^hb^_*p*_ is the averaged strength of the three base pairing hydrogen bonds. The {a–b}^m^ list is identified according to the procedure delineated earlier (*cf.*Fig. 2). Regarding the axial coupling of base stacking and ribose–phosphate backbone, rigidity at base *p* is based on the *k*^st^_*ij*_ and *k*^RP^_*ij*_ springs between the categorial atoms of *p* and *p* + 1 bases. Averaging over the springs in {a–b}^st^ and {a–b}^RP^ thus gives the base-stacking rigidity *k*^st^_*p*_ and backbone rigidity *k*^RP^_*p*_, respectively. As each base *p* has two sides of axial stacking, the 3′-side rigidity of base stacking is *k*^st^_*p*_ whereas the 5′-side value is *k*^st^_*p*−1_.

The mean inter-moiety rigidity, *k*^m^ (m = hb, BR, st, or RP), is the average of the *k*^m^_*p*_ values over bases, Fig. 3a. To understand the inter-atom couplings of inter-moiety rigidity, the strengths of atom pairs in {a–b}^m^ are averaged over the bases as the *k*^m^_a–b_ values. For example, given the {a–b}^hb^ list of mechanical hotspots in polyA base pairing, {C2–O2, N1–N3, N6–O4}, *k*^hb^_*p*_ reports their averaged hydrogen bonding strength at base *p*, while *k*^hb^_C2–O2_, *k*^hb^_N1–N3_, and *k*^hb^_N6–O4_ are the specific inter-atom strengths in the base pairing, Fig. 3b. Based on the canonical B-form structure of dsDNA, which is well maintained in the all-atom MD simulations of the transcription regulatory sequences studied here, proximity of atom pairs to grooves^64^ is employed to indicate their relative positions in the double helix. In the base pairing of polyA, the minor-groove side, middle, and major-groove side hydrogen bonding strengths are *k*^hb^_C2–O2_, *k*^hb^_N1–N3_, and *k*^hb^_N6–O4_, respectively, Fig. 3b.

## 3 Results and discussion

With the haENM spring constants quantified by structure-mechanics statistical learning, the profiles of inter-moiety rigidity are cross-compared to reveal the mechanical patterning in the DNA sequence motifs of transcription regulation. To understand the molecular origin, we look into the *k*^hb^_a–b_, *k*^st^_a–b_, and *k*^RP^_a–b_ values of mechanical hotspots (atom names a and b) in *k*^hb^_*p*_, *k*^st^_*p*_, and *k*^RP^_*p*_, respectively (*cf.*Fig. 3). The inter-moiety rigidity of each category is the average of the exceptionally strong spring constants, and the atoms that they connect are defined as mechanical hotspots, Fig. 3 and ESI.† The base-to-backbone chemomechanical linkage and their inter-atom coupling strengths are shown to provide an intuitive understanding for many experimental observables, including the persistence length and a variety of structural properties.

### 3.1 Compensatory competition between base pairing (transverse) and base stacking (axial)

**Fig. 4:**
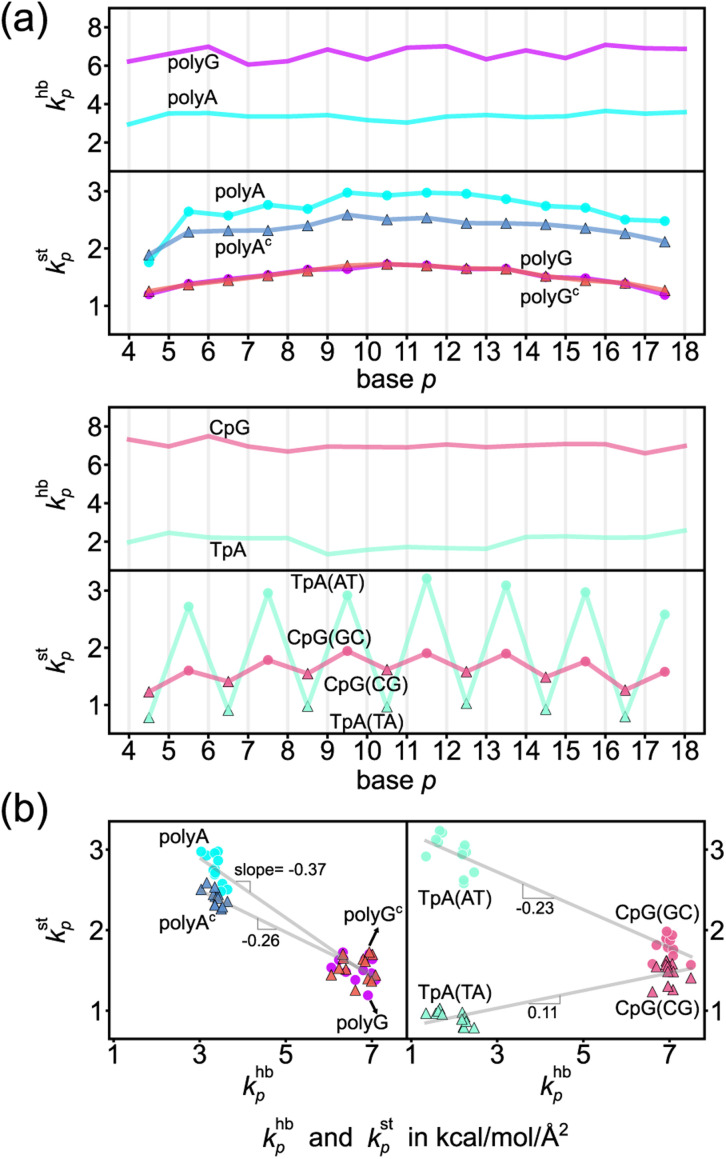
Base-pairing rigidity and base-stacking rigidity in the transcription regulatory sequences studied here. (a) *k*^hb^_*p*_ and *k*^st^_*p*_ along the sequence index *p*. Terminal bases are not included to discard the fraying effects.^65^ The shorthand notation hb is for base pairing hydrogen bonding and st is for base stacking. Top: The profiles of polyA and polyG. Bottom: The profiles of TpA and CpG. (b) The *k*^st^_*p*_–*k*^hb^_*p*_ plots of polyA, polyA^c^, polyG, and polyG^c^ (left) and of TpA(AT), TpA(TA), CpG(GC) and CpG(CG) (right). The linear best fit of *k*^st^_*p*_ to *k*^hb^_*p*_ is shown for each group of (polyA, polyG), (polyA^c^, polyG^c^), (TpA(AT), CpG(GC)), and (TpA(TA), CpG(CG)).

The *k*^hb^_*p*_-*versus-p* curve of polyG is about 1.8 times higher than that of polyA as one would expect for G–C pairing being stronger, Fig. 4a. The weaker A–T pairing, however, has much stronger rigidity in base stacking. The *k*^st^_*p*_–*k*^hb^_*p*_ plots of polyA and polyG and those of polyA^c^ and polyG^c^ illustrate their negative correlation in Fig. 4b. Plotting the 3′-side stacking rigidity *k*^st^_*p*−1_ with *k*^hb^_*p*_ gives a similar result, Fig. S15.† Even when considering the *k*^st^_*p*_ values in one of the dsDNA systems without referencing to those of another chain, the detailed analysis as reported in the ESI text and Fig. S18–S21† illustrates the specific competition between the base-stacking interaction and the base-pairing interaction. To understand this reciprocal relationship, we examine the inter-atom coupling strengths of the mechanical hotspots in *k*^st^_*p*_ and *k*^hb^_*p*_, *i.e.*, the *k*^st^_a–b_ and *k*^hb^_a–b_ values (*cf.*Fig. 3).

**Fig. 5:**
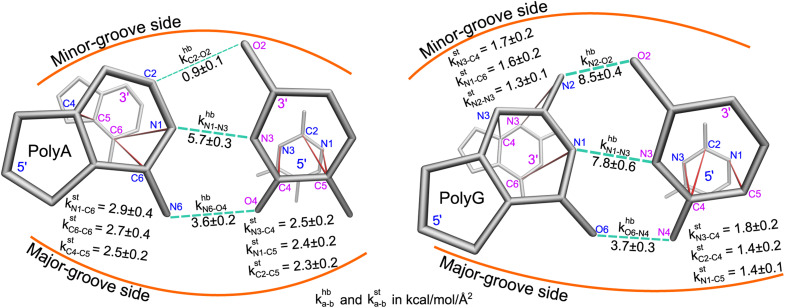
The strengths of inter-atom couplings in base-pairing rigidity and in base-stacking rigidity of polyA (left) and polyG (right), *i.e.*, their *k*^hb^_a–b_ and *k*^st^_a–b_ values. For base-pairing rigidity, the *k*^hb^_a–b_ values of atom pairs in the {a–b}^hb^ list of mechanical hotspots are displayed around the green dotted lines. For the {a–b}^st^ list of base-stacking rigidity, the 5′-side atoms are in blue and 3′-side atoms are in magenta.

Regarding the specific strength of hydrogen bonding, the weaker A–T pairing has the property of *k*^hb^_N6–O4_ at the major-groove side ≫ *k*^hb^_C2–O2_ at the minor-groove side, while the stronger G–C pairing has the opposite trend of *k*^hb^_O6–N4_ at the major-groove side < *k*^hb^_N2–O2_ at the minor-groove side, Fig. 5. A–T and G–C base pairing thus have distinct relative strengths in hydrogen bonding between the minor-groove side and the major-groove side. A–T and G–C base pairing exhibiting opposite relative strengths over groove sides was not noticed previously to the best of our knowledge. Furthermore, the mechanical coupling of base stacking is also similarly patterned. Fig. 5 shows that the polyA mechanical hotspots of *k*^st^_*p*_ lean over the major-groove side while those in polyG bias toward the minor-groove side. The mechanical hotspots of base stacking are thus consistent with the groove-side imbalance in base pairing, and the negative correlation of *k*^st^_*p*_ (axial) with *k*^hb^_*p*_ (transverse, *cf.*Fig. 1b) indicates compensatory competition between the axial base-stacking interaction with the transverse base-pairing interaction.

The base-stacking mechanical hotspots leaning toward the groove side of the stronger base-pairing hydrogen bonding, though, is not apparent in polyA^c^ and polyG^c^ of the smaller bases, Fig. 5. The strand-specific base-stacking rigidity can be seen in the distinctively higher *k*^st^_*p*_ values in polyA than those in polyA^c^. The much lower values of *k*^st^_*p*_ in polyG and polyG^c^, however, are nearly identical under the stronger base pairing, Fig. 4a.

The finding of compensatory competition between the transverse base pairing interaction and the axial base stacking interaction provides mechanistic insight for the sequence specificity observed in experimental DNA structures, such as the higher structural flexibility around the GG dinucleotide,^66,67^ which can be understood as the base stacking being weaker due to the stronger base pairing, similar to the case of low base-stacking rigidity in polyG. On the other side of the same coin, this mechanism explains the observation of AA and TT having maximal base overlaps in experimental structures,^68^ which gives rise to the stronger axial coupling in polyA. Our approach thus identifies a common molecular origin for a variety of sequence-dependent flexibilities observed in different structural analysis. As discussed in the ESI text with Movies S1 and S2,† base-stacking rigidity negatively correlating with base-pairing strength shows specific structural dynamics in the all-atom MD trajectories, including the wider distributions of slide and shift in polyG (Fig. S16†). Another prominent mechanical property due to the compensatory competition between base pairing and base stacking as reported earlier is the base-stacking hotspots leaning toward the major-groove side in polyA but not in polyA^c^, and with such difference in the axial couplings of the base pair, a large mean propeller twist of −11.8° is observed in the all-atom MD trajectory, Fig. S17.† This unique structural feature was also observed in the X-ray structures of polyA,^68–70^ and our analysis of inter-moiety rigidity provides the previously unknown chemical basis. With the nearly identical and much weaker base stacking in polyG and polyG^c^, a significantly smaller propeller twist (−3.4°) is observed instead, Fig. S17.†

### 3.2 Axially alternating rigidity of base stacking in ambigram sequences

The two strands in TpA and in CpG have identical, axially alternating sequences, and showcase how the chemical-scale mechanical mechanisms—the groove side-dependent strength of base pairing and the compensatory competition between the transverse base-pairing interaction and the axial base-stacking interaction—manifest under the ambigram symmetry. The *k*^hb^_*p*_-*versus-p* curves of base pairing in TpA and CpG display flat profiles of robust mechanical signals, Fig. 4a. A–T pairing in TpA and G–C pairing in CpG also exhibit distinct relative strengths between the minor-groove side hydrogen bonding and the major-groove side interaction (Fig. S12†), and this difference between the two types of complementarity is consistently observed as in the homopolymeric systems (Fig. 5). Furthermore, A–T pairing in TpA is weaker than that in polyA, Fig. 4a. This result is consistent with the NMR analysis showing that the strength of A–T base pairing depends on the axially-stacked neighbors.^71^ Our result shows that such behavior is also observed in G–C base pairing, and CpG *k*^hb^_*p*_ being noticeably higher than that in polyG is opposite to the ambigram-*versus*-homopolymeric comparison of A–T pairing, Fig. 4a.

With the two strands having the same sequence, the base-stacking mechanical coupling is specifically patterned in the structure of TpA and CpG. In both dsDNA, the base-stacking mechanical hotspots leaning over the groove side of the stronger base-pairing hydrogen bond is observed at the purine–pyrimidine dinucleotide of both strands but not at the pyrimidine–purine unit (Fig. S12†). The two strands in TpA indeed display identical *k*^st^_*p*_-*versus-p* profiles with drastic ups and downs in Fig. 4a, indicating that TpA(AT) has much stronger base stacking than TpA(TA) does. With mechanical hotspots of the dinucleotide unit and its complement having the same bias, the *k*^st^_*p*_ of TpA(AT) is even higher than that of polyA, Fig. 4a. However, the groove side-specific interactions under ambigram symmetry lead to poor base stacking at TpA(TA) (Fig. S12†) and its *k*^st^_*p*_ is ∼3 times lower, Fig. 4a. Drastic difference in base-stacking rigidity between the axially alternating dinucleotide units is a unique property of TpA. The *k*^st^_*p*_ of CpG has a similar up-and-down profile, but the difference between CpG(GC) and CpG(CG) in *k*^st^_*p*_ is much milder under the stronger base pairing, Fig. 4a. The compensatory competition between the axial base-stacking interaction with the transverse base-pairing interaction in terms of the negative correlation of *k*^st^_*p*_ with *k*^hb^_*p*_ is also observed based on the stronger base stacking of CpG(GC) and TpA(AT), Fig. 4b.

CpG has a unique property that the base-stacking mechanical hotspots leaning over the minor-groove side is also observed at the smaller cytosine in addition to guanine (Fig. S12†). In the other dsDNA sequences of TpA, polyA, and polyG studied here, on the contrary, only the mechanical coupling of the purine base is patterned in this manner. As such, the rigidity of both base stacking and base pairing in CpG are higher than those in polyG. This behavior is exceptional because the mechanical coupling exhibits negative correlation between the base-stacking rigidity and the base-pairing strength in the other cases. As discussed in the following, CpG also has a peculiar mechanical property in the backbone rigidity.

With base-pairing and base-stacking rigidity showing specific sequence patterning, how would mechanical coupling in backbone exhibit different behaviors is analyzed next. As discussed in the ESI text with Movies S3 and S4,† backbone structural dynamics in all-atom MD trajectories seem to relate to base-mediated interactions. The rigidity of the base-to-ribose linkage indeed shows intricate connection, Fig. S22.† In the following, we focus on the rigidity of the ribose—phosphate backbone, *k*^RP^_*p*_, which reveals the specific property in each sequence, especially the relative population of backbone conformation in the BI or BII state.

### 3.3 Backbone polymorphism is linked to base-mediated mechanical couplings

**Fig. 6:**
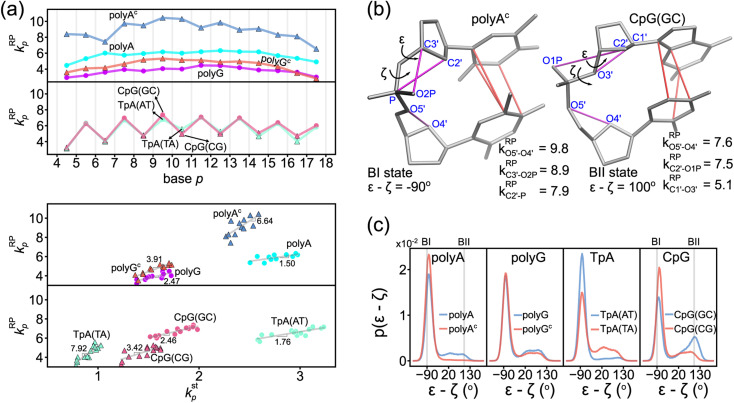
Rigidity of ribose-phosphate (RP) backbone has positive correlation with the base-stacking rigidity. (a) The *k*^RP^_*p*_-*versus-p* profiles of polyA, polyA^c^, polyG, polyG^c^, TpA, and CpG (top). The *k*^RP^_*p*_–*k*^st^_*p*_ plot for the transcription regulatory sequences (bottom). Grey lines are their linear best fits. (b) Illustration of backbone conformation in polyA^c^ at BI state (left) and in CpG(GC) at BII state (right). The mechanical hotspots in {a–b}^RP^ (magenta) and {a–b}^st^ (red) are listed. BI and BII states are defined by the difference between *ε* and *ζ* dihedral angles of the backbone. All rigidities are in kcal mol^−1^ Å^−2^. (c) The probability density distribution of *ε*–*ζ* in the 5 μs all-atom MD trajectory of the DNA sequence motifs.

The phosphodiester backbone of dsDNA is an important protein binding site. The *k*^RP^_*p*_-*versus-p* profiles in Fig. 6a illustrate that this moiety indeed has sequence-specific rigidity patterns for molecular recognition. A common behavior is the positive correlation of backbone rigidity *k*^RP^_*p*_ with base-stacking rigidity *k*^st^_*p*_, and the DNA sequence motifs studied here exhibit various extents of correlation, bottom panel in Fig. 6a.

To analyze if an alternative backbone conformation is involved, mechanical hotspots in the {a–b}^RP^ list of *k*^RP^_*p*_ provide key information. For the exceptionally high *k*^RP^_*p*_ values in polyA^c^ (Fig. 6a), mechanical hotspots that deliver the very high *k*^RP^_a–b_ values signal the BI state of the dsDNA conformation, which is defined by the *ε* and *ζ* backbone dihedral angles,^72^Fig. 6b. Indeed, the high backbone rigidity of polyA^c^ is found to have an *ε*–*ζ* distribution of ultra-high BI state population in the all-atom MD trajectory, Fig. 6c. The higher BI-state population in T-rich dsDNA was also noticed in NMR analysis and X-ray structures,^73,74^ but the mechanical origin was unclear. The significantly lower *k*^RP^_*p*_ values of backbone rigidity in polyA, on the other hand, indicates a noticeable BII-state population, which is typical behavior of B-form dsDNA.^75^ The backbone mechanical hotspots in the two polyA strands are thus different atom pairs, Fig. S14.† The above results exemplify that the peculiar structural features in dsDNA would exhibit specific chemical-scale mechanical properties, which can be captured by our quantification of inter-moiety rigidity.

The backbone conformation revealing specific mechanical hotspots is also seen in TpA. TpA(AT) with the much higher backbone rigidity is similar to polyA^c^ in terms of the mechanical hotspots (Fig. S14†) and the high BI-state population (Fig. 6c). TpA(TA) that has lower backbone rigidity instead has the polyA-like mechanical hotspots and a similar BII-state population. Recall that the base-stacking rigidity of TpA(AT) is also much stronger than that of TpA(TA) (*cf.*Fig. 4a). Overall, backbone conformation around thymine being more populated in the BI state correlates with the higher backbone rigidity and the stronger base stacking in polyA^c^ and in TpA(AT). The other non-thymine cases that have a similar BII-state population and lower backbone rigidity include polyG, polyG^c^, and CpG(CG) (Fig. 6c), and they have identical mechanical hotspots (Fig. S14†). This result further illustrates that consistent mechanical signals can be captured for the specific backbone conformation.

Another peculiar behavior in backbone rigidity is observed in CpG. Both the *k*^st^_*p*_ and *k*^RP^_*p*_ values of CpG(GC) are higher than those of CpG(CG) and polyG, Fig. 6a. It turns out that CpG(GC) has backbone mechanical hotspots signaling the BII-state conformation, Fig. 6b, and has a significantly higher BII-state population than the other cases, Fig. 6c. This feature of the backbone conformation around the GC dinucleotide was noticed in crystal structures and NMR signals,^76^ but the connection to chemical-scale mechanical properties was not recognized. The backbone conformation around CpG guanine being more populated in the BII state is shown here to correlate with the higher rigidity of backbone coupling and base stacking.

The chemical-scale mechanical picture revealed from the analysis of the inter-moiety rigidities in dsDNA is the groove side-specific strength of base pairing (Fig. 5), the compensatory competition between the transverse base-pairing interaction and the axial base-stacking interaction (Fig. 4b), and the backbone rigidity *k*^RP^_*p*_ correlating with the base-stacking rigidity *k*^st^_*p*_ (Fig. 6a). To test the sensitivity of these behaviors to molecular mechanical energetics, a different force field (OL15)^77^ with the refinements dedicated to the structurally important torsional angles in DNA is used to conduct all-atom MD simulations for the rigidity-graph analysis. Despite the different representations of intra-molecular interactions, similar values of chemical-scale rigidities are obtained for the DNA systems of different sequences. The compensatory competition between the axial base-stacking interaction and the transverse base-pairing interaction as well as the positive correlation of backbone rigidity and base-stacking rigidity are consistently observed. The OL15 slopes in *k*^hb^_*p*_–*k*^st^_*p*_ and *k*^RP^_*p*_–*k*^st^_*p*_ plots (Fig. S23 and S24†) are also quantitatively similar to those presented here. The robust mechanistic behaviors of chemical-scale rigidities may not be surprising since these force fields were developed with common objectives of reproducing the available data on DNA structures. To understand such diverse sequence-specific behaviors in genomic regulation, including the force-field dependence of structural dynamics, the framework developed here for quantifying the inter-moiety rigidities provides a way to learn about the chemical-scale mechanical origin.

### 3.4 Mechanical code as the trigram of base-to-backbone rigidity

**Fig. 7:**
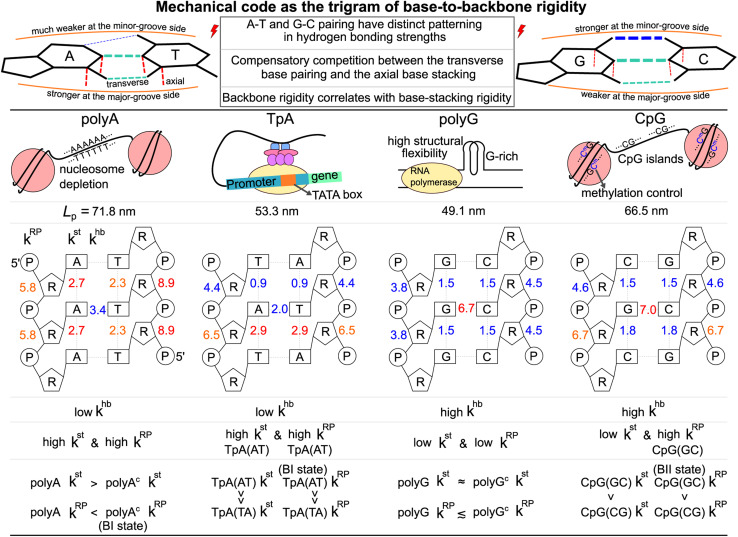
Mechanical code as the trigram of base-to-backbone rigidity. Top: A–T and C–G base pairing (transverse) has distinct relative strength between the major-groove side hydrogen bonding and the minor-groove side hydrogen bonding. Mechanical codes of the transcription regulatory sequences indicate compensatory competition between the axial base-stacking interaction and the transverse base-pairing interaction, as well as positive correlation of the backbone rigidity with the base-stacking strength (both axial). Middle: Biological functions of the transcription regulatory sequences. The persistence length *L*_p_ calculated from the all-atom MD of each system is listed. Bottom: The trigram of base-to-backbone rigidity for polyA, polyG, TpA, and CpG in terms of the *k*^hb^, *k*^st^ and *k*^RP^ values in kcal mol^−1^ Å^−2^. The peculiar behaviors of different systems are specified. These properties show that while a larger-scale material property such as persistence length may be similar, each dsDNA has a unique mechanical code.

The aforementioned results can be summarized by the mean rigidity between chemical moieties, *i.e.*, averaging the *k*^m^_*p*_ of bases to *k*^m^ (*cf.*Fig. 3 and S25†). The base-to-backbone inter-moiety rigidity as the value of *k*^hb^, *k*^st^, and *k*^RP^ appears as a unique trigram in each of the polyA, polyG, TpA, and CpG sequences, and could be viewed as a mechanical code, Fig. 7. This property provides the missing information of chemical-scale mechanics for experimental observables on a larger scale such as persistence length that tend to have limited sensitivity to sequence variation.^78–82^ Our quantitative analysis of inter-moiety rigidity as presented above demonstrates that the chemical-scale mechanical properties indeed have sequence-sensitive behaviors.

Since the processes measured in experiments are composed of changes in inter-moiety distances, mechanical code can facilitate the development of mechanistic understanding. Taking the bending of dsDNA as an example, the atomic structure of the double helix (*cf.*Fig. 1b) suggests that the inter-moiety distances along the axial directions are likely perturbed to greater extents. Inter-base distances were indeed shown to have higher relevance to persistence length, while the inter-atom distances of base pairing and backbone have lower but still significant influence on *L*_p_.^22^ Therefore, despite the lower *k*^hb^ in transverse base pairing, ultra-long *L*_p_ can be achieved in polyA through high *k*^st^ and high *k*^RP^ (Fig. 7). The large bending resistance^11–13^ to wrap around histone proteins was shown to lead to the high occurrence of the polyA sequence in nucleosome depletion regions where the transcription starts in the chromosome.^28–32^ As a contrasting case, one may also ask: Why does polyG have significantly lower *L*_p_ than polyA despite the stronger base-pairing interaction? This property is related to the high structural flexibility observed in the G-rich repressor segment of a promoter^33,34^ and is shown here to associate with the low *k*^st^ and low *k*^RP^ of polyG.

CpG having a rather long *L*_p_ closer to the value of polyA is illustrated here to arrive from a different trigram with high *k*^hb^ and high *k*^RP^, Fig. 7. CpG islands play key roles in the initiation of mammal gene expression.^38–40^ This result is a first illustration that the different trigrams of base-to-backbone rigidity would have similar mechanical properties on a larger scale. Another example is the similarly short *L*_p_ values of TpA and polyG exhibiting different mechanical codes, Fig. 7. For gene expression in eukaryotic and archaeal cells, the key function of binding RNA polymerase II is enabled by the TATA box upper stream of the transcription initiation site.

For base-pair geometries such as propeller twist, slide, and shift that are discussed earlier, our quantification of base-to-backbone inter-moiety rigidity offers a useful platform for analysis since specifically patterned mechanical coupling can be identified for the different behaviors in structural properties. For example, CpG dsDNA displaying polymorphism as the BI or BII state^74^ and polyA^c^ showing an ultra-high BI-state population^73^ are shown to exhibit specific mechanical properties in backbone, and the strength of which correlates with the base-stacking rigidity. The sequence-specific mechanical codes summarized in Fig. 7 also suggest that TpA would have kinkable behaviors as observed in single-molecule experiments^13^ and in X-ray structures.^43^

## 4 Conclusion

For the DNA sequence motifs involved in various regulatory processes of transcription, we ask: Could there be a mechanical code in them that illustrates the sequence-dependent properties of deformation akin to the form of the genetic code? This question is addressed in this article by quantifying the rigidity between base, ribose, and backbone moieties, and collating those that are statistically significant. Our results show that indeed each sequence motif has a unique trigram of base-to-backbone rigidity which could be taken as a form of mechanical code. An immediate consequence of note is that while different dsDNA sequences may exhibit indistinguishable macroscopic mechanical bendability, they are fully resolvable on the mechanical-code level. The predicted rigidity differences reflect the relative structural flexibilities at the chemical-moiety level, which can potentially be verified using such experimental approaches as X-ray structural biology^43^ and NMR spectroscopy.^73,83^

These results also imply DNA sequence-dependent mechanical signal transduction—DNA mechanical allostery^84–86^—where local mechanical deformation in dsDNA may impact on gene-regulation actions at remote sites. Under this premise, it follows, it would be of great interest to understand the elements that make up the DNA mechanical code as well as the basic principles of constructing it. Since the DNA mechanical code is based on physical interactions, we expect such an understanding to be generalizable beyond the sequences studied here. For example, in addition to the conventional wisdom that G–C is the stronger base pairing compared to A–T, the two types of complementarity are shown here to have opposite relative strengths between the minor-groove side hydrogen bonding and the major-groove side interaction. The mechanical coupling of base stacking is also shown to pattern in a similar way as the imbalance in base-pairing interactions (*cf.*Fig. 1b). These behaviors, in combination with the negative correlation between *k*^st^_*p*_ and *k*^hb^_*p*_ values (Fig. 4b) indicate compensatory competition between the axial base-stacking interaction with the transverse base-pairing interaction, which is the most discriminating feature of the mechanical code. Another prominent property is the positive correlation of backbone *k*^RP^_*p*_ with *k*^st^_*p*_ (Fig. 6b). The mechanistic understanding of mechanical codes also provides an intuitive physical picture for the specific structural features seen in experiments, including such base-pairing geometries as propeller twist^68–70^ and the relative BI or BII state population of the backbone,^73,74^ because it clarifies how different structural behaviors result from the mechanical properties at the chemical moiety level.

The dsDNA sequence–structure–dynamics–function relationship is one of the guiding principles that have helped defining the field as it continues to evolve. Along this line of thinking, however, it remains unclear as to how DNA functional mechanics could be understood using notions from biochemistry, structural biology, or bioinformatics. The mechanical code concept described here represents the first step outside of, yet complementing, the current paradigm and provides a fresh new way of thinking about this relationship. Thus, in addition to systematic studies to fully decipher the mechanical code, immediate possibilities to further explore this concept include DNA allostery, protein–DNA interactions, and DNA binding drugs. Beyond application to biological systems, one may envision the chemical principles of mechanical-code construction to be potentially very useful in advancing smart biomimetic devices, for example, by DNA origami.

## Data availability

The data underlying this article are available in FigShare at https://dx.doi.org/10.6084/m9.figshare.21828906.

## Author contributions

YTC: conceptualization, methodology, software, writing – original draft preparation; HY: conceptualization, writing – review & editing; JWC: conceptualization, methodology, software, writing – original draft preparation, writing – review & editing, supervision.

## Conflicts of interest

There are no conflicts to declare.

## Supplementary Material

SC-014-D3SC01671D-s001

SC-014-D3SC01671D-s002

SC-014-D3SC01671D-s003

SC-014-D3SC01671D-s004

SC-014-D3SC01671D-s005
