# Supplementary material for: Mechanical codes of chemical-scale specificity in DNA motifs
Source: Chem Sci. 2023 Aug 29;14(37):10155–66. doi: 10.1039/d3sc01671d (PMC10529945; doi:10.1039/d3sc01671d)
Supplement: SC-014-D3SC01671D-s005 [file SC-014-D3SC01671D-s005.pdf]

Electronic Supplementary Material (ESI) for Chemical Science.
This journal is © The Royal Society of Chemistry 2023

Supporting Information: Mechanical Codes of Chemical-Scale Specificity in DNA Motifs

Yi-Tsao Chen,† Haw Yang,‡ and Jhih-Wei Chu*,¶

†*Institute of Bioinformatics and Systems Biology, National Yang Ming Chiao Tung University, Hsinchu 30010, Taiwan*

‡*Department of Chemistry, Princeton University, Princeton, NJ 08544, USA*

¶*Institute of Bioinformatics and Systems Biology, Department of Biological Science and Technology, and Institute of Molecular Medicine and Bioengineering, National Yang Ming Chiao Tung University, Hsinchu 30010, Taiwan*

E-mail: jwchu@nctu.edu.tw

Methods

Determining the persistence length from dynamics of dsDNA

To quantify the bending rigidity from dsDNA dynamics, we model a dsDNA molecule as an elastic rod, which is approximated by the helical axis of dsDNA. In this work, Curves+[S1] is employed to find the helical axis for each atomistic configurations in MD trajectories. The representative of each base pair (denoted as $\mathbf{r}_i$, $i = 1$ to 21) on the elastic rod can be obtained through the discretization of the helical axis, and the arc length of the elastic rod, $s(\mathbf{r}_i)$, arises naturally as a function of $\mathbf{r}_i$. To avoid the fraying effect, the starting point of the rod is set to $\mathbf{r}_4$ so that $s(\mathbf{r}_4) = 0$, and the end point of the rod is set to $\mathbf{r}_{18}$. The contour

length of the rod, L, thereby can be expressed as

$$L = \int_{\mathbf{r}_4}^{\mathbf{r}_{18}} |\frac{d\mathbf{r}(s)}{ds}| ds. \tag{1}$$

Next, the local tangent vector at the ith base pair to the rod is approximated by

$$\mathbf{t}_i = \frac{\mathbf{r}_{i+1} - \mathbf{r}_i}{|\mathbf{r}_{i+1} - \mathbf{r}_i|}, \tag{2}$$

and the shape of the rod at the ith base pair, $\theta(s(\mathbf{r}_i))$, can be calculated by

$$\theta(s(\mathbf{r}_i)) = \arccos(\mathbf{t}_i \cdot \mathbf{t}_4) \quad i = 4 \text{ to } 18. \tag{3}$$

Furthermore, the shape $\theta(s)$ can be expressed as the superposition of Fourier modes

$$\theta(s) = \sqrt{\frac{2}{L}} \sum_{n=0}^{\infty} a_n \cos(\frac{n\pi s}{L}) \tag{4}$$

where the nth mode amplitude

$$a_n = \sqrt{\frac{2}{L}} \int_0^L \theta(s) \cos(\frac{n\pi s}{L}) ds. \tag{5}$$

In the assumption of the elastic rod, the energy of bending, $\mathcal{U}$, is a quadratic sum of the mode amplitudes

$$\mathcal{U} = \int_0^L \frac{1}{2} EI(\frac{d\theta}{ds} - \frac{d\theta^0}{ds})^2 \cdot s = \frac{1}{2} EI \sum_{n=1}^{\infty} (\frac{n\pi}{L})^2 (a_n - a_n^0)^2, \tag{6}$$

where $\theta^0(s)$ is the shape in the absence of external forces, a_n^0 is the amplitude of the mode in the absence of an external force and EI is the flexural rigidity. According to the equipartition

principle, each mode has $\frac{1}{2}k_{\mathrm{B}}T$ of thermal energy in it, so that

$$\mathrm{Var}(a_n) = \langle a_n^2 \rangle - \langle a_n^0 \rangle = \frac{k_{\mathrm{B}}T}{EI}(\frac{L}{n\pi})^2 = \frac{1}{L_{\mathrm{p}}}(\frac{L}{n\pi})^2, \tag{7}$$

where $\mathrm{Var}(a_n)$ is the variance of the nth mode amplitude and L_{p} is the persistence length. Therefore, the calculation of $\mathrm{Var}(a_n)$ from MD trajectory, together with the knowledge of the contour length, provides an estimate of L_{p}.[S2] Here, the mode index n is set to 2 for the reason that the wavelength of $n = 2$ is consistent with the length scale of the bending deformation at the whole molecule level.

Categorization of rigidity graphs

Base-pairing hydrogen bond (hb) The rigidity graph $\mathbf{K}_n^{\mathrm{hb}}$ is aimed at characterizing the strengths of the canonical Watson-Crick base pairing for the trajectory window n. The vertices and edges of each A-T base pair are selected based on the heavy atoms of the two hydrogen bonds in addition to the C2-O2 atomtype pair at the minor-groove side, as shown in Figure 2. For each G-C base pair, the vertices and edges are selected based on the heavy atoms of the three hydrogen bonds. The edge weights of $\mathbf{K}_n^{\mathrm{hb}}$ are the corresponding spring constants k_{ij}^{hb} of the window n. The selection of the list of most strongly coupled atomtype pairs $\{\text{a-b}\}^{\mathrm{hb}}$ is trivial, i.e., $\{\text{C2-O2}, \text{N1-N3}, \text{N6-O4}\}$ for the A-T base pair and $\{\text{N2-O2}, \text{N1-N3}, \text{O6-N4}\}$ for the G-C base pair.

Base-stacking (st) The rigidity graph $\mathbf{K}_n^{\mathrm{st}}$ is designed to capture the strengths of stacking interaction for the trajectory window n. The vertices comprise all nucleobase atoms in the two strands of dsDNA, and the edges are the springs in between adjacent nucleobases defined in the haENM. The edge weights of $\mathbf{K}_n^{\mathrm{st}}$ are the corresponding spring constants k_{ij}^{RP} of the window n.

Base-ribose (BR) The rigidity graph $\mathbf{K}_n^{\mathrm{BR}}$ is designed to capture the coupling strengths between the nucleobase and ribose in a nucleotide for the trajectory window n. The vertices comprise the six atoms of ribose (C1', C2', C3', C4', O4', O3') and all heavy atoms of nucleobase of all nucleotides in the two strands of dsDNA, and the edges are those haENM springs defined in between the nucleobase and the ribose for each nucleotide. Notice that the atom pairs coinciding with covalent bonds and bond angles are excluded. The edge weights of $\mathbf{K}_n^{\mathrm{BR}}$ are the corresponding spring constants k_{ij}^{BR} of the window n.

Ribose-phosphate (RP) The rigidity graph $\mathbf{K}_n^{\mathrm{RP}}$ is designed to quantify the rigidity of the ribose-phosphate backbone in the two strands of dsDNA for the trajectory window n. The vertices comprise the atoms of all riboses and phosphate groups in dsDNA, and the edges are the connecting springs between a ribose and a phosphate group. Notice that the atom pairs coinciding with covalent bonds and bond angles are excluded. The edge weights of $\mathbf{K}_n^{\mathrm{RP}}$ are the corresponding spring constants k_{ij}^{RP} of the window n.

The correlation between base-pairing rigidity and base-stacking rigidity in the transcription regulatory sequences

To understand how each coupling strength of $\{\text{a-b}\}^{\mathrm{hb}}$ correlates with those of $\{\text{a-b}\}^{\mathrm{st}}$, a detailed analysis is performed for the transcription regulatory sequences, Figures S18-S21. At the level of inter-atom couplings for each base p, the strength of each atom pair in the $\{\text{a-b}\}^{\mathrm{st}}$ list of stacking hotspots is plotted with the strength of each base pairing hydrogen bond. Despite the noisier data, each inter-atom coupling of stacking shows negative correlation with all three base-pairing interactions in polyA, Figure S18a. For polyG, negative stacking-base pairing correlation in strength is observed at the major-groove side for the weakest hydrogen bond, Figure S19a. At base-middle where the hydrogen bond is about twice stronger, the stacking strength is almost independent of the base pairing strength. At the minor-groove

side of the highest-strength hydrogen bond in polyG, the more than five times weaker stacking becomes positively correlated with the hydrogen bonding strength. Consistent with the behavior observed in polyA, both base-stacking rigidity of TpA(AT) and TpA(TA) have negative correlation with the base-pairing rigidity, Figure S20a. Within the CpG strands, the prominent stacking strengths have positive correlation with the minor-groove side hydrogen bonding spring constant as in polyG, while negative correlation with those at base middle and the major-groove side is also observed, Figure S21a. These results corroborate the tight connection of stacking with base-pairing interactions and the ordering of hydrogen bonding strength across grooves casts significant influence on base stacking.

Movie S1 Axial-transverse competition in polyA and polyG (top view). The persistent hydrogen bonds in polyG come with movements parallel to the base-pairing plane and such transverse displacements would disrupt the base stacking. With the very weak A-T pairing at the minor-grove side, alternative base movements of more intact stacking toward the major-groove side are observed. In the larger base strand of polyA, movements along the adenine plane are clearly more restrained when comparing to the guanine strand of polyG, and the slide and shift of polyG indeed have wider distributions than those of polyA, Figure S16.

Movie S2 Axial-transverse competition in polyA and polyG (side view).

Movie S3 Structural dynamics of backbone and base-stacking in polyA and polyG. The backbone of polyA^c exhibits the slightest fluctuation and stay in the BI state, which is consistent with the exceptionally high k_p^{RP} values in polyA^c. On the other hand, the backbones of polyA, polyG and polyG^c exhibit large fluctuation and have frequent transitions between the BI state and the BII state. In the cases of polyG and polyG^c, strong base pairing makes the bases move more frequently, thereby leading to the large fluctuation in their backbones.

Movie S4 Structural dynamics of backbone and base-stacking in TpA and CpG. The backbone dynamics of TpA(AT) is similar to the one of polyA^c, which tends to being the BI state. The TpA(TA) backbone, on the other hand, is similar to polyA with a noticeable BII population. In CpG, it can be noticed that the backbone of CpG(GC) stays in the BII state for a longer time, but the backbone of CpG(CG) prefers to stay in the BI state.

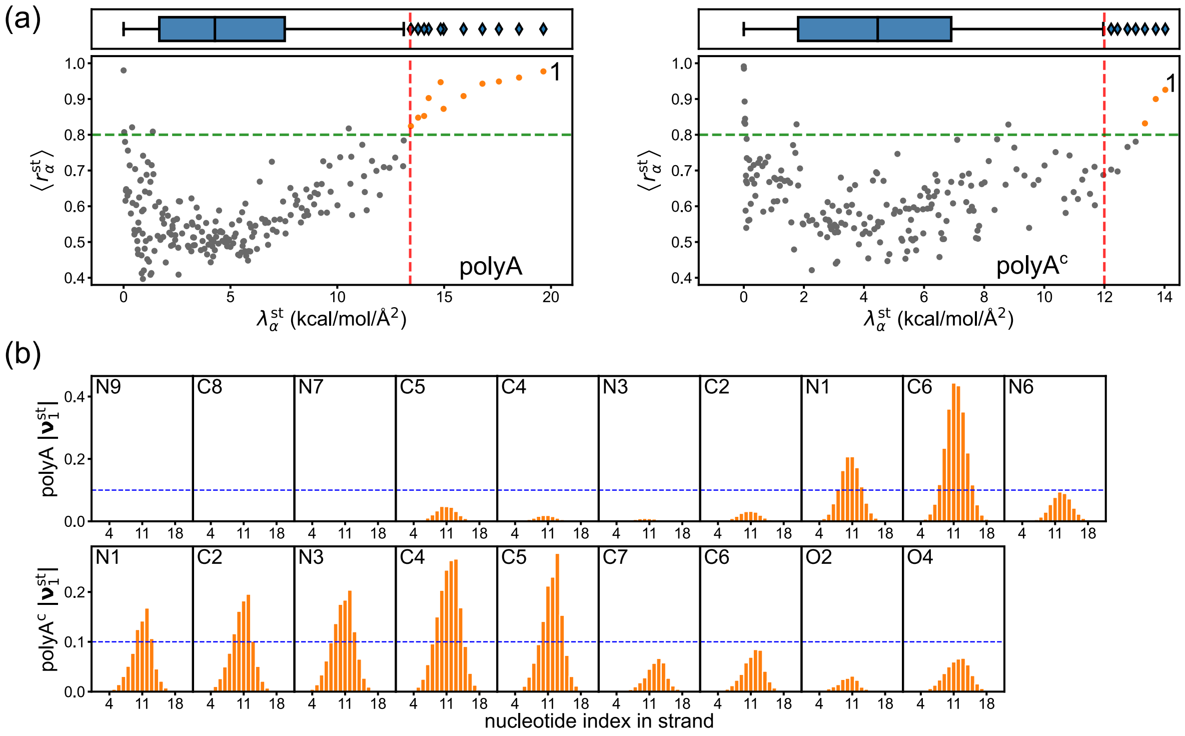

Figure S1: Identification of prominent modes in $\overline{\mathbf{K}}^{st}$ of polyA and $\overline{\mathbf{K}}^{st}$ of polyA^c. (a) The distributions of averaged mean-mode content $\langle r_\alpha^{st} \rangle$ and eigenvalue λ_α^{st}, where α is the index of mode. Statistical outliers of the λ_α^{st} distribution ($\lambda_\alpha^{st} > 1.5$ IQR, the red dotted line) that also have high mean-mode contents ($\langle r_\alpha^{st} \rangle > 0.8$, green dotted line) are identified as the prominent modes of $\overline{\mathbf{K}}^{st}$, as the orange dots shown. (b) For each atom in polyA and polyA^c, the magnitude of the αth prominent mode, $|\boldsymbol{\nu}_\alpha^{st}|$, quantifies the importance of the atom for $\overline{\mathbf{K}}^{st}$. For the purpose of illustration, $|\boldsymbol{\nu}_1^{st}|$ of polyA and $|\boldsymbol{\nu}_1^{st}|$ of polyA^c are shown. Each separate bar chart shows $|\boldsymbol{\nu}_1^{st}|$ for a specific atom in base A (polyA) or base T (polyA^c) along the nucleotide index. The high weight atoms of $\overline{\mathbf{K}}^{st}$ are atoms whose $|\boldsymbol{\nu}_\alpha^{st}|$ are higher than 0.1 (blue dotted lines).

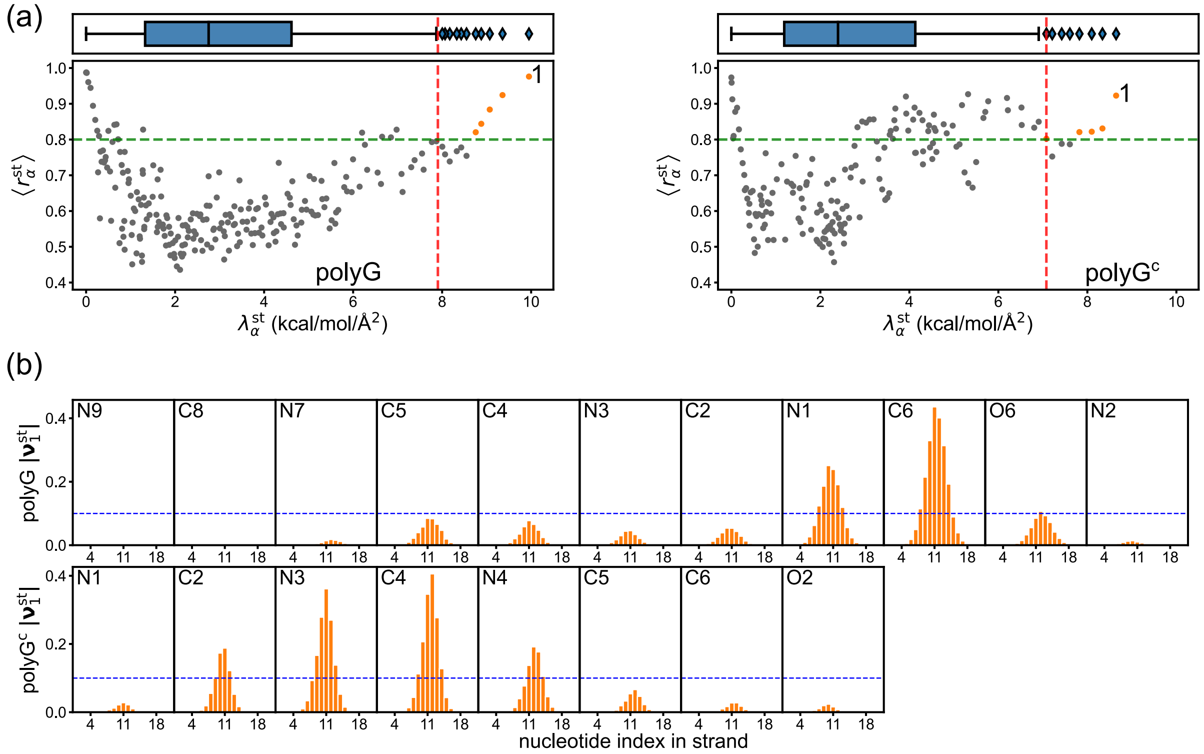

Figure S2: Identification of prominent modes in $\overline{\mathbf{K}}^{\text{st}}$ of polyG and $\overline{\mathbf{K}}^{\text{st}}$ of polyG^{c}. (a) The distributions of averaged mean-mode content $\langle r_\alpha^{\text{st}} \rangle$ and eigenvalue $\lambda_\alpha^{\text{st}}$, where α is the index of mode. Statistical outliers of the $\lambda_\alpha^{\text{st}}$ distribution ($\lambda_\alpha^{\text{st}} > 1.5$ IQR, the red dotted line) that also have high mean-mode contents ($\langle r_\alpha^{\text{st}} \rangle > 0.8$, green dotted line) are identified as the prominent modes of $\overline{\mathbf{K}}^{\text{st}}$, as the orange dots shown. (b) For each atom in polyG and polyG^{c}, the magnitude of the αth prominent mode, $|\boldsymbol{\nu}_\alpha^{\text{st}}|$, quantifies the importance of the atom for $\overline{\mathbf{K}}^{\text{st}}$. For the purpose of illustration, $|\boldsymbol{\nu}_1^{\text{st}}|$ of polyG and $|\boldsymbol{\nu}_1^{\text{st}}|$ of polyG^{c} are shown. Each separate bar chart shows $|\boldsymbol{\nu}_1^{\text{st}}|$ for a specific atom in base G (polyG) or base C (polyG^{c}) along the nucleotide index. The high weight atoms of $\overline{\mathbf{K}}^{\text{st}}$ are atoms whose $|\boldsymbol{\nu}_\alpha^{\text{st}}|$ are higher than 0.1 (blue dotted lines).

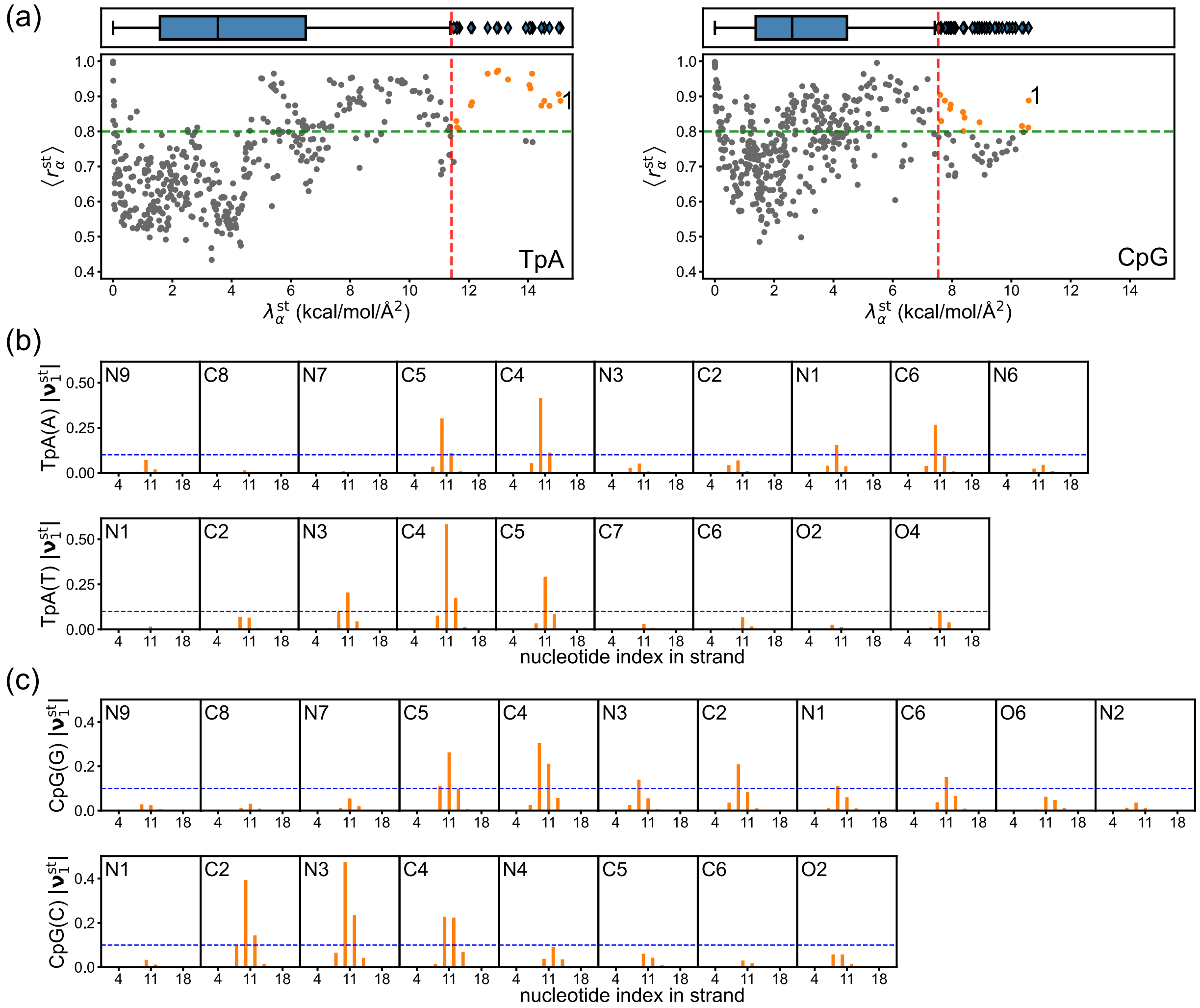

Figure S3: Identification of prominent modes in $\overline{\mathbf{K}}^{st}$ of TpA and $\overline{\mathbf{K}}^{st}$ of CpG. (a) The distributions of averaged mean-mode content $\langle r_\alpha^{st} \rangle$ and eigenvalue λ_α^{st}, where α is the index of mode. Statistical outliers of the λ_α^{st} distribution ($\lambda_\alpha^{st} > 1.5$ IQR, the red dotted line) that also have high mean-mode contents ($\langle r_\alpha^{st} \rangle > 0.8$, green dotted line) are identified as the prominent modes of $\overline{\mathbf{K}}^{st}$, as the orange dots shown. For each atom in TpA and CpG, the magnitude of the αth prominent mode, $|\boldsymbol{\nu}_\alpha^{st}|$, quantifies the importance of the atom for $\overline{\mathbf{K}}^{st}$. For the purpose of illustration, (b) $|\boldsymbol{\nu}_1^{st}|$ of TpA and (c) $|\boldsymbol{\nu}_1^{st}|$ of CpG are shown. Each separate bar chart shows $|\boldsymbol{\nu}_1^{st}|$ for a specific atom in base A (TpA(A)), base T (TpA(A)), base G (CpG(G)) or base C (CpG(C)) along the nucleotide index. The high weight atoms of $\overline{\mathbf{K}}^{st}$ are atoms whose $|\boldsymbol{\nu}_\alpha^{st}|$ are higher than 0.1 (blue dotted lines).

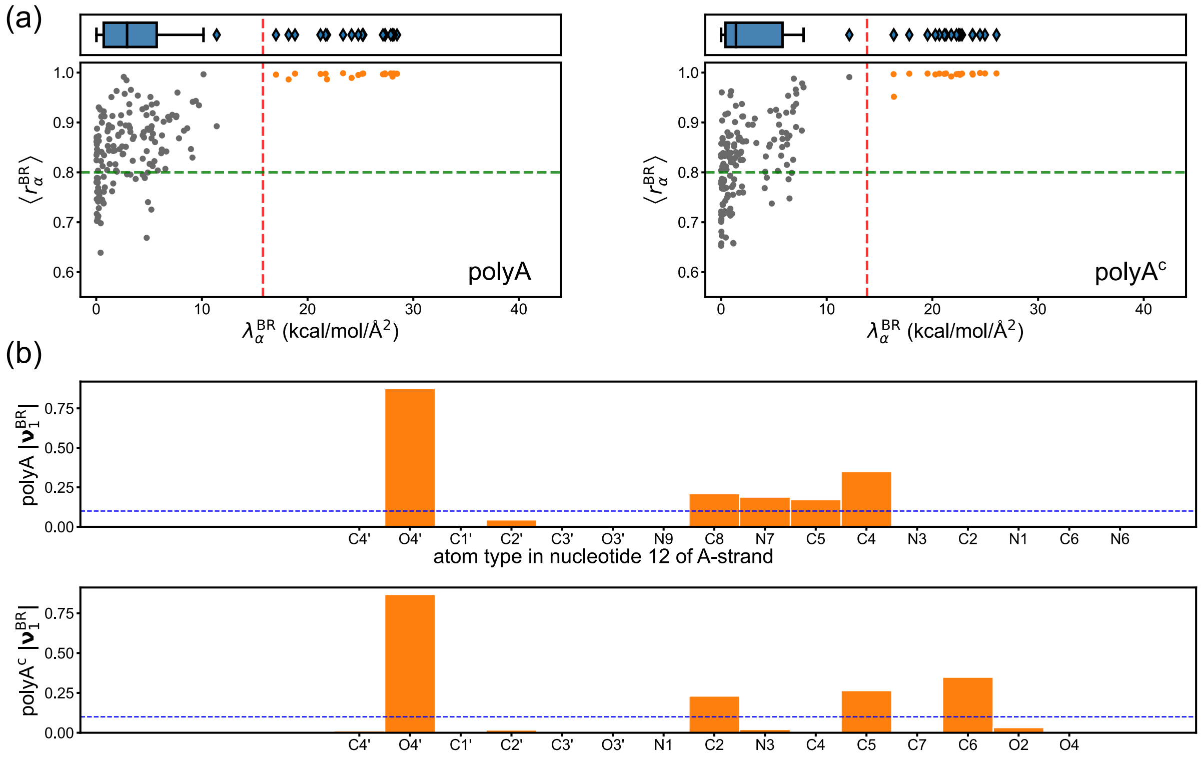

Figure S4: Identification of prominent modes in $\overline{\mathbf{K}}^{\mathrm{BR}}$ of polyA and $\overline{\mathbf{K}}^{\mathrm{BR}}$ of polyA^c. (a) The distributions of averaged mean-mode content $\langle r_\alpha^{\mathrm{BR}} \rangle$ and eigenvalue $\lambda_\alpha^{\mathrm{BR}}$, where α is the index of mode. Statistical outliers of the $\lambda_\alpha^{\mathrm{BR}}$ distribution ($\lambda_\alpha^{\mathrm{BR}} > 1.5$ IQR, the red dotted line) that also have high mean-mode contents ($\langle r_\alpha^{\mathrm{BR}} \rangle > 0.8$, green dotted line) are identified as the prominent modes of $\overline{\mathbf{K}}^{\mathrm{BR}}$, as the orange dots shown. (b) For each atom in polyA and polyA^c, the magnitude of the αth prominent mode, $|\boldsymbol{\nu}_\alpha^{\mathrm{BR}}|$, quantifies the importance of the atom for $\overline{\mathbf{K}}^{\mathrm{BR}}$. For the purpose of illustration, $|\boldsymbol{\nu}_1^{\mathrm{BR}}|$ of polyA and $|\boldsymbol{\nu}_1^{\mathrm{BR}}|$ of polyA^c are shown. The bar chart shows $|\boldsymbol{\nu}_1^{\mathrm{BR}}|$ for the atoms of ribose and the atoms of base (polyA: A, polyA^c: T). The high weight atoms of $\overline{\mathbf{K}}^{\mathrm{BR}}$ are atoms whose $|\boldsymbol{\nu}_\alpha^{\mathrm{BR}}|$ are higher than 0.1 (blue dotted lines).

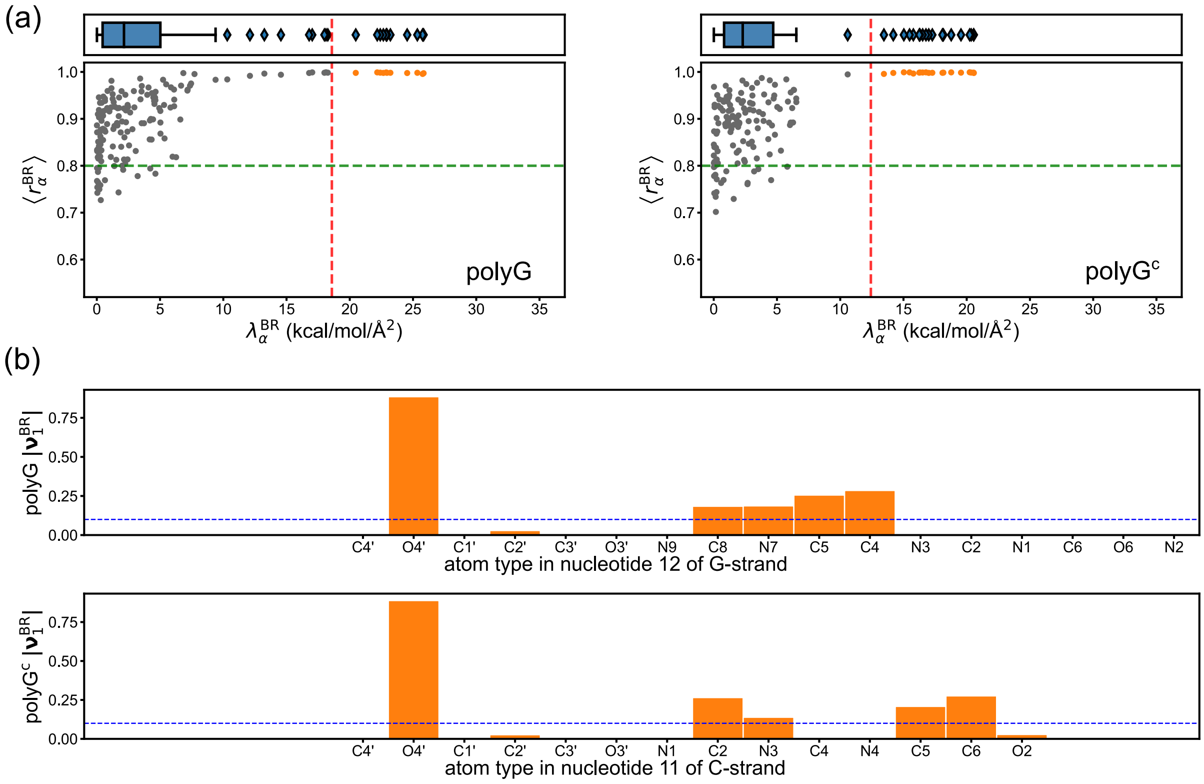

Figure S5: Identification of prominent modes in $\overline{\mathbf{K}}^{\mathrm{BR}}$ of polyG and $\overline{\mathbf{K}}^{\mathrm{BR}}$ of polyG^{c}. (a) The distributions of averaged mean-mode content $\langle r_\alpha^{\mathrm{BR}} \rangle$ and eigenvalue $\lambda_\alpha^{\mathrm{BR}}$, where α is the index of mode. Statistical outliers of the $\lambda_\alpha^{\mathrm{BR}}$ distribution ($\lambda_\alpha^{\mathrm{BR}} > 1.5$ IQR, the red dotted line) that also have high mean-mode contents ($\langle r_\alpha^{\mathrm{BR}} \rangle > 0.8$, green dotted line) are identified as the prominent modes of $\overline{\mathbf{K}}^{\mathrm{BR}}$, as the orange dots shown. (b) For each atom in polyG and polyG^{c}, the magnitude of the αth prominent mode, $|\boldsymbol{\nu}_\alpha^{\mathrm{BR}}|$, quantifies the importance of the atom for $\overline{\mathbf{K}}^{\mathrm{BR}}$. For the purpose of illustration, $|\boldsymbol{\nu}_1^{\mathrm{BR}}|$ of polyG and $|\boldsymbol{\nu}_1^{\mathrm{BR}}|$ of polyG^{c} are shown. The bar chart shows $|\boldsymbol{\nu}_1^{\mathrm{BR}}|$ for the atoms of ribose and the atoms of base (polyG: G, polyG^{c}: C). The high weight atoms of $\overline{\mathbf{K}}^{\mathrm{BR}}$ are atoms whose $|\boldsymbol{\nu}_\alpha^{\mathrm{BR}}|$ are higher than 0.1 (blue dotted lines).

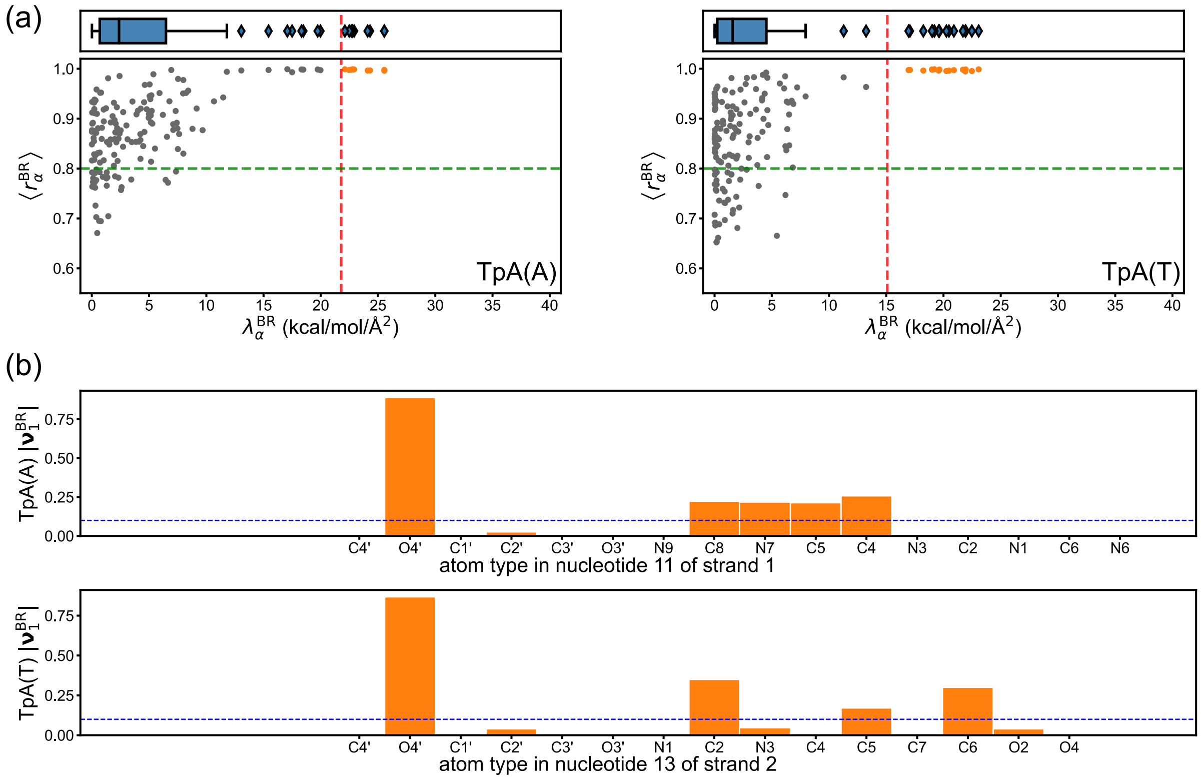

Figure S6: Identification of prominent modes in $\overline{\mathbf{K}}^{\mathrm{BR}}$ of TpA(A) and $\overline{\mathbf{K}}^{\mathrm{BR}}$ of TpA(T). (a) The distributions of averaged mean-mode content $\langle r_\alpha^{\mathrm{BR}} \rangle$ and eigenvalue $\lambda_\alpha^{\mathrm{BR}}$, where α is the index of mode. Statistical outliers of the $\lambda_\alpha^{\mathrm{BR}}$ distribution ($\lambda_\alpha^{\mathrm{BR}} > 1.5$ IQR, the red dotted line) that also have high mean-mode contents ($\langle r_\alpha^{\mathrm{BR}} \rangle > 0.8$, green dotted line) are identified as the prominent modes of $\overline{\mathbf{K}}^{\mathrm{BR}}$, as the orange dots shown. (b) For each atom in TpA(A) and TpA(T), the magnitude of the αth prominent mode, $|\boldsymbol{\nu}_\alpha^{\mathrm{BR}}|$, quantifies the importance of the atom for $\overline{\mathbf{K}}^{\mathrm{BR}}$. For the purpose of illustration, $|\boldsymbol{\nu}_1^{\mathrm{BR}}|$ of TpA(A) and $|\boldsymbol{\nu}_1^{\mathrm{BR}}|$ of TpA(T) are shown. The bar chart shows $|\boldsymbol{\nu}_1^{\mathrm{BR}}|$ for the atoms of ribose and the atoms of base (TpA(A): A, TpA(T): T). The high weight atoms of $\overline{\mathbf{K}}^{\mathrm{BR}}$ are atoms whose $|\boldsymbol{\nu}_\alpha^{\mathrm{BR}}|$ are higher than 0.1 (blue dotted lines).

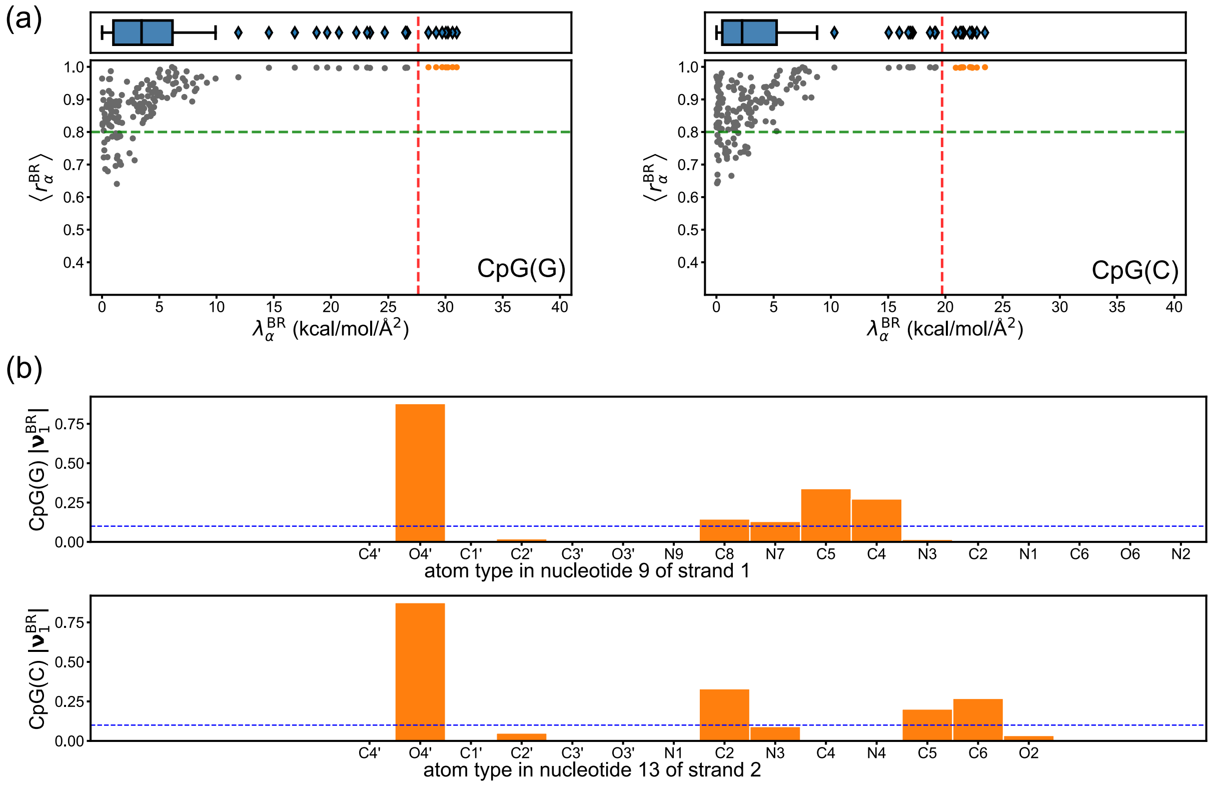

Figure S7: Identification of prominent modes in $\overline{\mathbf{K}}^{\mathrm{BR}}$ of CpG(G) and $\overline{\mathbf{K}}^{\mathrm{BR}}$ of CpG(C). (a) The distributions of averaged mean-mode content $\langle r_\alpha^{\mathrm{BR}} \rangle$ and eigenvalue $\lambda_\alpha^{\mathrm{BR}}$, where α is the index of mode. Statistical outliers of the $\lambda_\alpha^{\mathrm{BR}}$ distribution ($\lambda_\alpha^{\mathrm{BR}} > 1.5$ IQR, the red dotted line) that also have high mean-mode contents ($\langle r_\alpha^{\mathrm{BR}} \rangle > 0.8$, green dotted line) are identified as the prominent modes of $\overline{\mathbf{K}}^{\mathrm{BR}}$, as the orange dots shown. (b) For each atom in CpG(G) and CpG(C), the magnitude of the αth prominent mode, $|\boldsymbol{\nu}_\alpha^{\mathrm{BR}}|$, quantifies the importance of the atom for $\overline{\mathbf{K}}^{\mathrm{BR}}$. For the purpose of illustration, $|\boldsymbol{\nu}_1^{\mathrm{BR}}|$ of CpG(G) and $|\boldsymbol{\nu}_1^{\mathrm{BR}}|$ of CpG(C) are shown. The bar chart shows $|\boldsymbol{\nu}_1^{\mathrm{BR}}|$ for the atoms of ribose and the atoms of base (CpG(G): G, CpG(C): C). The high weight atoms of $\overline{\mathbf{K}}^{\mathrm{BR}}$ are atoms whose $|\boldsymbol{\nu}_\alpha^{\mathrm{BR}}|$ are higher than 0.1 (blue dotted lines).

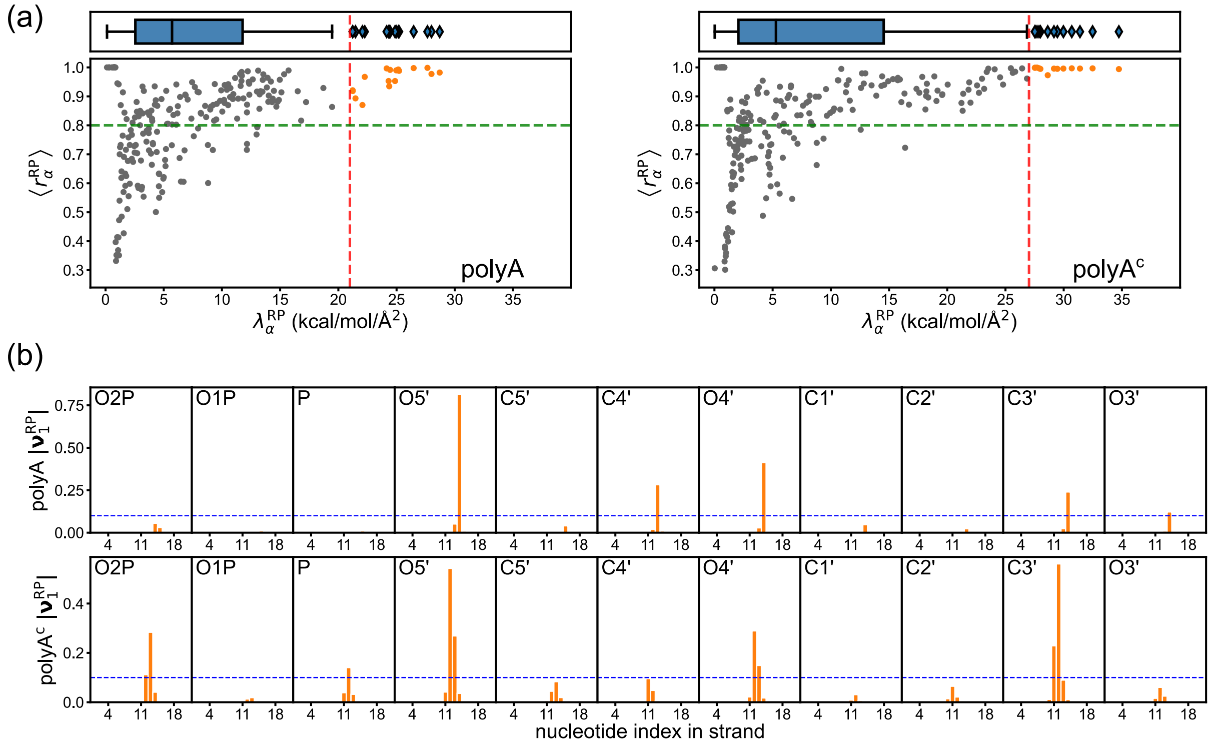

Figure S8: Identification of prominent modes in $\overline{\mathbf{K}}^{\mathrm{RP}}$ of polyA and $\overline{\mathbf{K}}^{\mathrm{RP}}$ of polyA^c. (a) The distributions of averaged mean-mode content $\langle r_\alpha^{\mathrm{RP}} \rangle$ and eigenvalue $\lambda_\alpha^{\mathrm{RP}}$, where α is the index of mode. Statistical outliers of the $\lambda_\alpha^{\mathrm{RP}}$ distribution ($\lambda_\alpha^{\mathrm{RP}} > 1.5$ IQR, the red dotted line) that also have high mean-mode contents ($\langle r_\alpha^{\mathrm{RP}} \rangle > 0.8$, green dotted line) are identified as the prominent modes of $\overline{\mathbf{K}}^{\mathrm{RP}}$, as the orange dots shown. (b) For each atom in polyA and polyA^c, the magnitude of the αth prominent mode, $|\boldsymbol{\nu}_\alpha^{\mathrm{RP}}|$, quantifies the importance of the atom for $\overline{\mathbf{K}}^{\mathrm{RP}}$. For the purpose of illustration, $|\boldsymbol{\nu}_1^{\mathrm{RP}}|$ of polyA and $|\boldsymbol{\nu}_1^{\mathrm{RP}}|$ of polyA^c are shown. Each separate bar chart shows $|\boldsymbol{\nu}_1^{\mathrm{RP}}|$ for a specific atom in ribose-phosphate backbone along the nucleotide index. The high weight atoms of $\overline{\mathbf{K}}^{\mathrm{RP}}$ are atoms whose $|\boldsymbol{\nu}_\alpha^{\mathrm{RP}}|$ are higher than 0.1 (blue dotted lines).

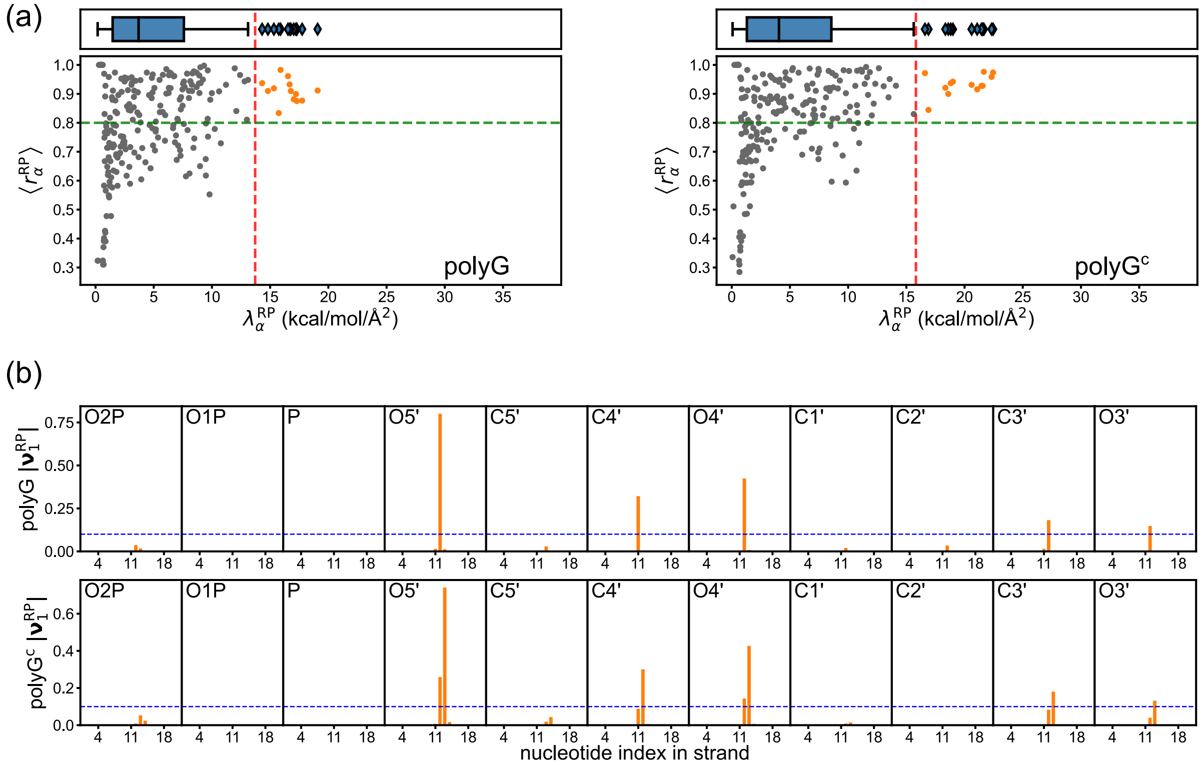

Figure S9: Identification of prominent modes in $\overline{\mathbf{K}}^{\mathrm{RP}}$ of polyG and $\overline{\mathbf{K}}^{\mathrm{RP}}$ of polyG^{c}. (a) The distributions of averaged mean-mode content $\langle r_\alpha^{\mathrm{RP}} \rangle$ and eigenvalue $\lambda_\alpha^{\mathrm{RP}}$, where α is the index of mode. Statistical outliers of the $\lambda_\alpha^{\mathrm{RP}}$ distribution ($\lambda_\alpha^{\mathrm{RP}} > 1.5$ IQR, the red dotted line) that also have high mean-mode contents ($\langle r_\alpha^{\mathrm{RP}} \rangle > 0.8$, green dotted line) are identified as the prominent modes of $\overline{\mathbf{K}}^{\mathrm{RP}}$, as the orange dots shown. (b) For each atom in polyG and polyG^{c}, the magnitude of the αth prominent mode, $|\boldsymbol{\nu}_\alpha^{\mathrm{RP}}|$, quantifies the importance of the atom for $\overline{\mathbf{K}}^{\mathrm{RP}}$. For the purpose of illustration, $|\boldsymbol{\nu}_1^{\mathrm{RP}}|$ of polyG and $|\boldsymbol{\nu}_1^{\mathrm{RP}}|$ of polyG^{c} are shown. Each separate bar chart shows $|\boldsymbol{\nu}_1^{\mathrm{RP}}|$ for a specific atom in ribose-phosphate backbone along the nucleotide index. The high weight atoms of $\overline{\mathbf{K}}^{\mathrm{RP}}$ are atoms whose $|\boldsymbol{\nu}_\alpha^{\mathrm{RP}}|$ are higher than 0.1 (blue dotted lines).

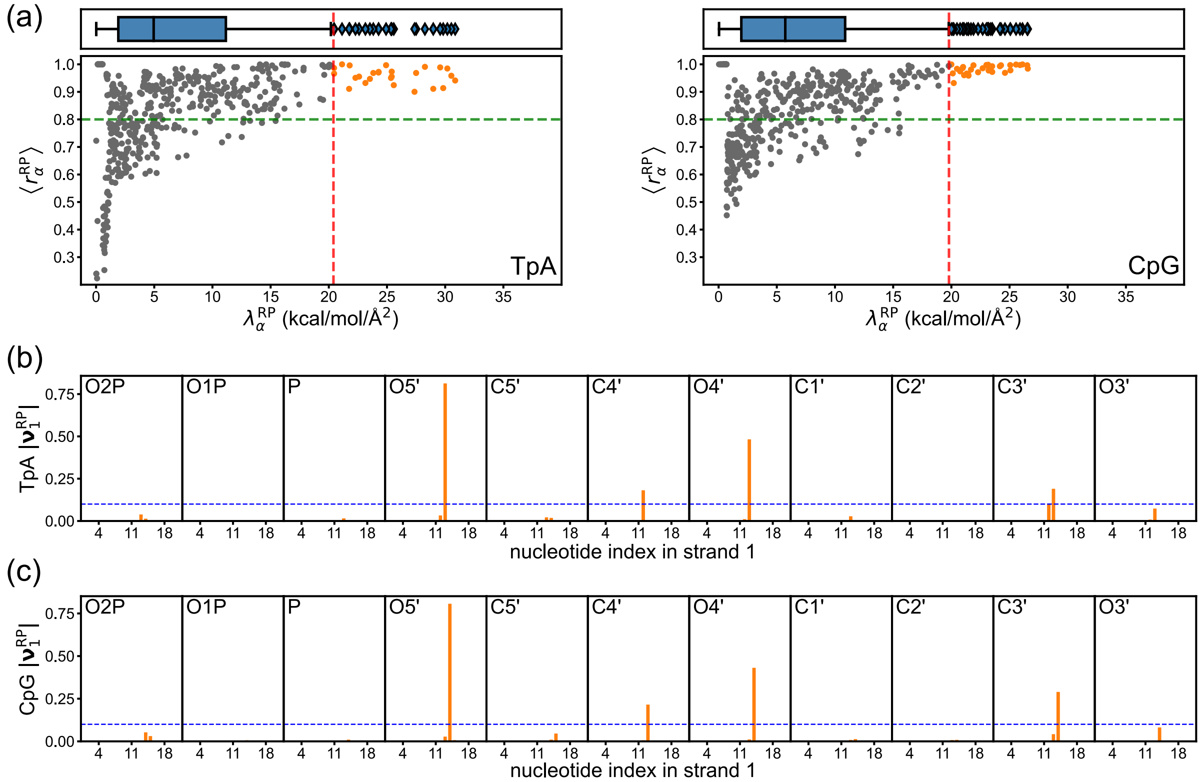

Figure S10: Identification of prominent modes in $\overline{\mathbf{K}}^{\text{RP}}$ of TpA and $\overline{\mathbf{K}}^{\text{RP}}$ of CpG. (a) The distributions of averaged mean-mode content $\langle r_\alpha^{\text{RP}} \rangle$ and eigenvalue $\lambda_\alpha^{\text{RP}}$, where α is the index of mode. Statistical outliers of the $\lambda_\alpha^{\text{RP}}$ distribution ($\lambda_\alpha^{\text{RP}} > 1.5$ IQR, the red dotted line) that also have high mean-mode contents ($\langle r_\alpha^{\text{RP}} \rangle > 0.8$, green dotted line) are identified as the prominent modes of $\overline{\mathbf{K}}^{\text{RP}}$, as the orange dots shown. For each atom in TpA and CpG, the magnitude of the αth prominent mode, $|\boldsymbol{\nu}_\alpha^{\text{RP}}|$, quantifies the importance of the atom for $\overline{\mathbf{K}}^{\text{RP}}$. For the purpose of illustration, (b) $|\boldsymbol{\nu}_1^{\text{RP}}|$ of TpA and (c) $|\boldsymbol{\nu}_1^{\text{RP}}|$ of CpG are shown. Each separate bar chart shows $|\boldsymbol{\nu}_1^{\text{RP}}|$ for a specific atom in ribose-phosphate backbone along the nucleotide index. The high weight atoms of $\overline{\mathbf{K}}^{\text{RP}}$ are atoms whose $|\boldsymbol{\nu}_\alpha^{\text{RP}}|$ are higher than 0.1 (blue dotted lines).

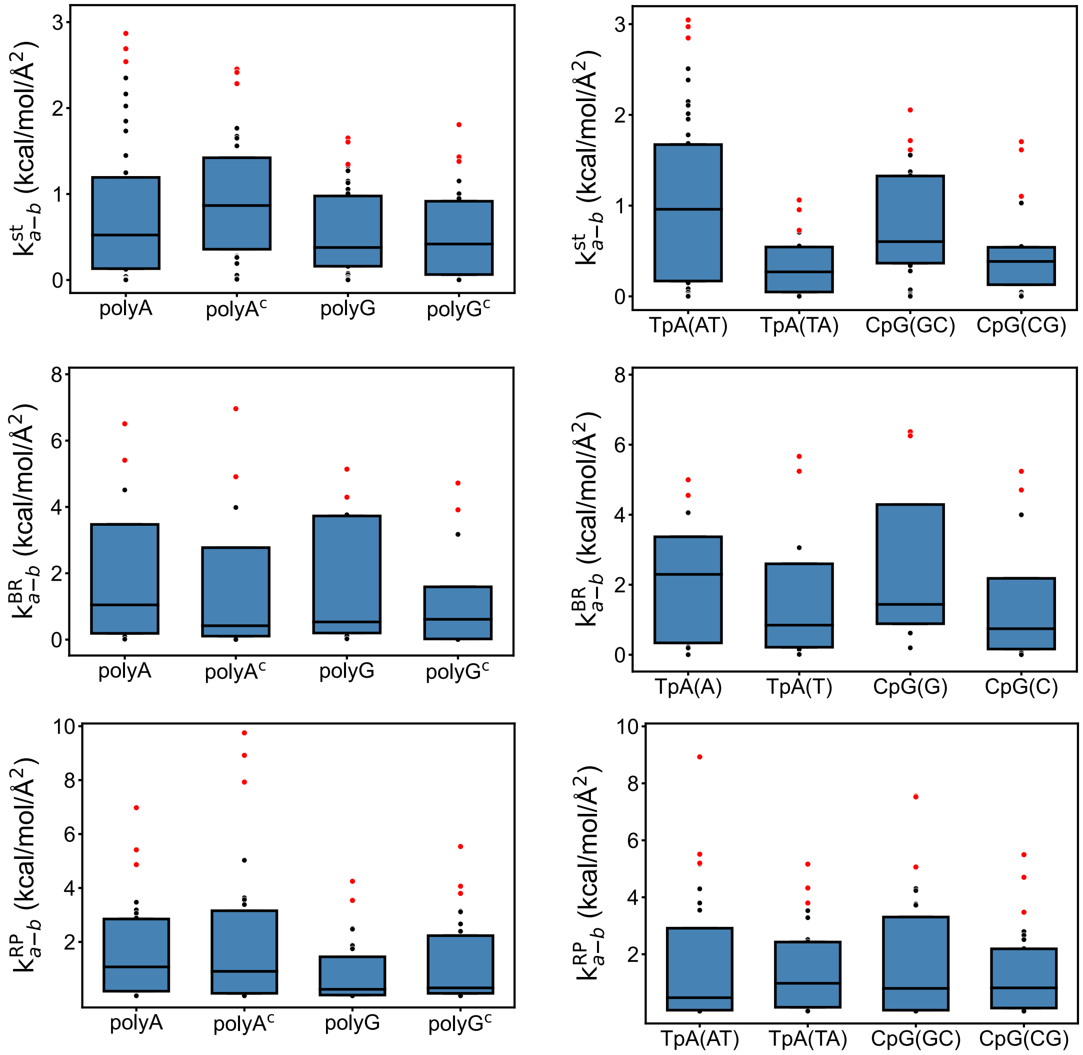

Figure S11: The distributions of $k^{m}_{a\text{-}b}$ for all types of base step (m=st,RP) or base (m=BR) shown as the boxplots. $k^{m}_{a\text{-}b}$ is the average of the corresponding k^{m}_{ij} of a particular atom pair *a*-*b* over all base steps or bases. The atom pairs whose $k^{m}_{a\text{-}b}$ is larger than Q3 are recognized as the outliers (dots). For m=st,RP, the atom pairs having the top three $k^{m}_{a\text{-}b}$ are selected as the most strongly atom pairs (red dots). For m=BR, the atom pairs having the top two $k^{m}_{a\text{-}b}$ are selected as the most strongly atom pairs (red dots).

(a)

polyA		polyA^c		polyG		polyG^c		TpA(AT)		TpA(TA)		CpG(GC)		CpG(CG)	
a − b	k^{st}_{a-b}	a − b	k^{st}_{a-b}	a − b	k^{st}_{a-b}	a − b	k^{st}_{a-b}	a − b	k^{st}_{a-b}	a − b	k^{st}_{a-b}	a − b	k^{st}_{a-b}	a − b	k^{st}_{a-b}
N1-C6	2.9	N3-C4	2.5	N3-C4	1.7	N3-C4	1.8	C4-C5	3.0	N1-N7	1.1	C4-C4	2.1	C2-C5	1.7
C6-C6	2.7	N1-C5	2.4	N1-C6	1.6	C2-C4	1.4	N1-N3	3.0	C2-C5	1.0	C2-C2	1.7	N3-C6	1.6
C4-C5	2.5	C2-C5	2.3	N2-N3	1.3	N1-C5	1.4	C6-C4	2.8	C4-N6	0.7	N9-C5	1.6	N1-N7	1.1

(b)

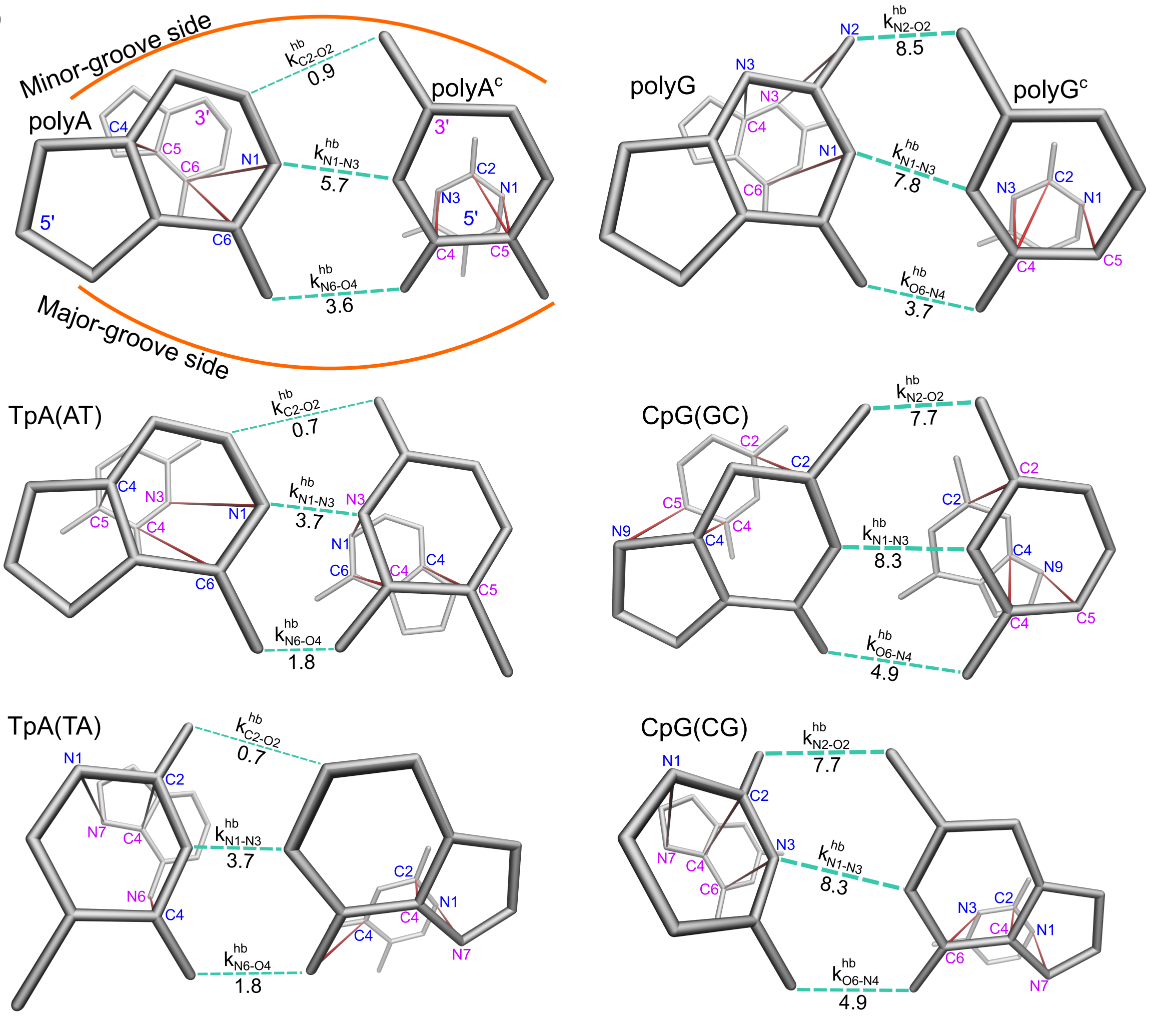

Figure S12: Most strongly coupled atom pairs $\{a\text{-}b\}^{st}$. (a) The list of the strength $k^{st}_{a\text{-}b}$ (kcal/mol/Å^2) for $\{a\text{-}b\}^{st}$ of all types of base step in the transcription regulatory sequences. The strength of a particular pair $k^{st}_{a\text{-}b}$ is the average of the corresponding k^{st}_{ij} over all central base steps. (b) The molecular illustrations of all pairs in $\{a\text{-}b\}^{st}$ (red lines). For the purpose of comparing the base-stacking rigidity with base-pairing rigidity, the strength $k^{hb}_{a\text{-}b}$ of atom pairs in $\{a\text{-}b\}^{hb}$ are also demonstrated (green dotted lines).

(a)

polyA		polyA^c		polyG		polyG^c		TpA(A)		TpA(T)		CpG(G)		CpG(C)	
a − b	k^{BR}_{a-b}	a − b	k^{BR}_{a-b}	a − b	k^{BR}_{a-b}	a − b	k^{BR}_{a-b}	a − b	k^{BR}_{a-b}	a − b	k^{BR}_{a-b}	a − b	k^{BR}_{a-b}	a − b	k^{BR}_{a-b}
O4'-C4	6.5	O4'-C6	7.0	O4'-C4	5.1	O4'-C6	4.7	O4'-C8	5.0	O4'-C6	5.7	O4'-C5	6.4	O4'-C6	5.2
O4'-C8	5.4	O4'-C5	4.9	O4'-C8	4.3	O4'-C2	3.9	O4'-C4	4.6	O4'-C2	5.2	O4'-C4	6.3	O4'-C2	4.7

(b)

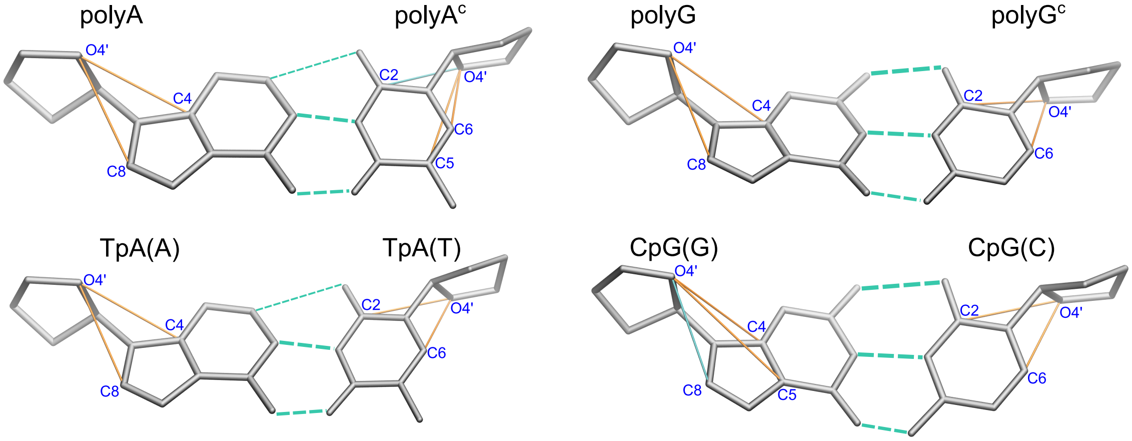

Figure S13: Most strongly coupled atom pairs $\{a\text{-}b\}^{BR}$. (a) The list of the strength $k^{BR}_{a\text{-}b}$ (kcal/mol/Å^2) for $\{a\text{-}b\}^{BR}$ of all types of base in the transcription regulatory sequences. The strength of a particular pair $k^{BR}_{a\text{-}b}$ is the average of the corresponding k^{BR}_{ij} over all central bases. (b) The molecular illustrations of all pairs in $\{a\text{-}b\}^{BR}$ (orange lines). For purine, the common pattern of $\{a\text{-}b\}^{BR}$ is {O4'-C4, O4'-C8}, but the O4'-C8 spring is not included in the $\{a\text{-}b\}^{BR}$ of CpG(G) (green solid line). In the case of pyrimidine, the general pattern of $\{a\text{-}b\}^{BR}$ is {O4'-C2, O4'-C6}, but the O4'-C2 spring is not included in the $\{a\text{-}b\}^{BR}$ of polyA^c (green solid line). For the purpose of showing the effect of base pairing on the pattern of BR mechanical hotspots, the atom pairs in $\{a\text{-}b\}^{hb}$ are also demonstrated (green dotted lines).

(a)

polyA		polyA^c		polyG		polyG^c		TpA(AT)		TpA(TA)		CpG(GC)		CpG(CG)	
a – b	k^{RP}_{a-b}	a – b	k^{RP}_{a-b}	a – b	k^{RP}_{a-b}	a – b	k^{RP}_{a-b}	a – b	k^{RP}_{a-b}	a – b	k^{RP}_{a-b}	a – b	k^{RP}_{a-b}	a – b	k^{RP}_{a-b}
O5'-O4'	7.0	O5'-O4'	9.8	O5'-O4'	4.2	O5'-O4'	5.5	O5'-O4'	8.9	O5'-O4'	5.2	O5'-O4'	7.6	C4'-O5'	5.5
C4'-O5'	5.4	C3'-O2P	8.9	O4'-O3'	3.6	C4'-O5'	4.1	O1P-C5'	5.5	C4'-O5'	4.3	C2'-O1P	7.5	O5'-O4'	4.7
O4'-O3'	4.9	C2'-P	7.9	C4'-O5'	3.5	O4'-O3'	3.8	C2'-P	5.2	C2'-O1P	3.8	C1'-O3'	5.1	O4'-O3'	3.5

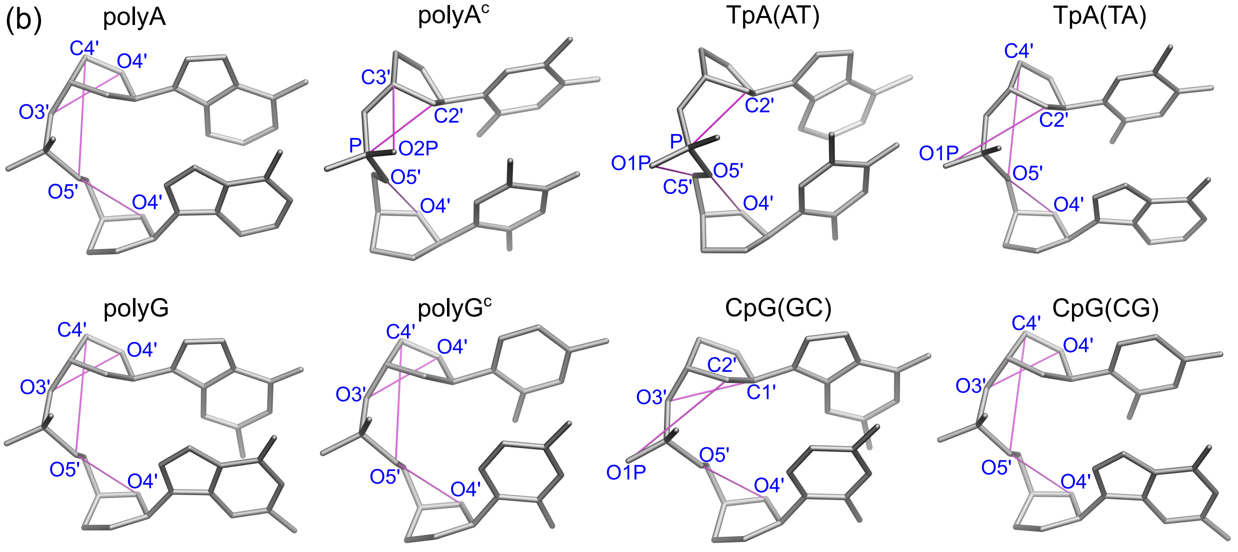

Figure S14: Most strongly coupled atom pairs $\{a\text{-}b\}^{RP}$. (a) The list of the strength $k^{RP}_{a\text{-}b}$ (kcal/mol/Å^2) for $\{a\text{-}b\}^{RP}$ of all types of base step in the transcription regulatory sequences. The strength of a particular pair $k^{RP}_{a\text{-}b}$ is the average of the corresponding k^{RP}_{ij} over all central base steps. (b) The molecular illustrations of all pairs in $\{a\text{-}b\}^{RP}$ (magenta lines).

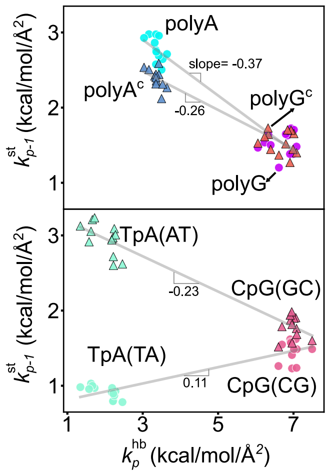

Figure S15: The $k^{st}_{p\text{-}1}$-k^{hb}_p plots of polyA, polyA^c, polyG and polyG^c (top) and of TpA(AT), TpA(TA), CpG(GC) and CpG(CG) (bottom). The linear best fit of $k^{st}_{p\text{-}1}$ to k^{hb}_p is shown for each group of (polyA, polyG), (polyA^c, polyG^c), (TpA(AT), CpG(GC)) and (TpA(TA), CpG(CG)).

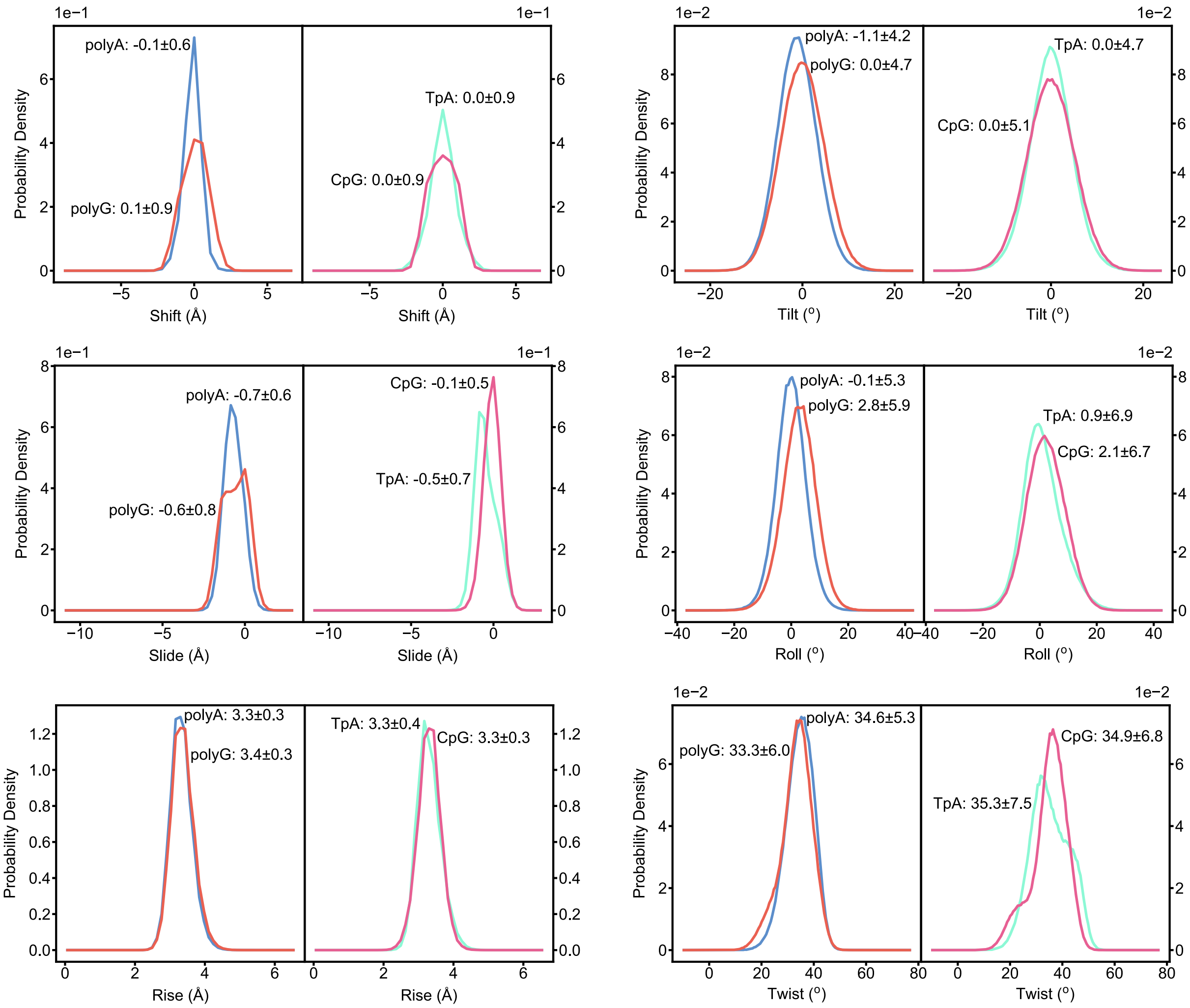

Figure S16: The characterization of base step parameters in the MD simulations of the transcription regulatory sequences.

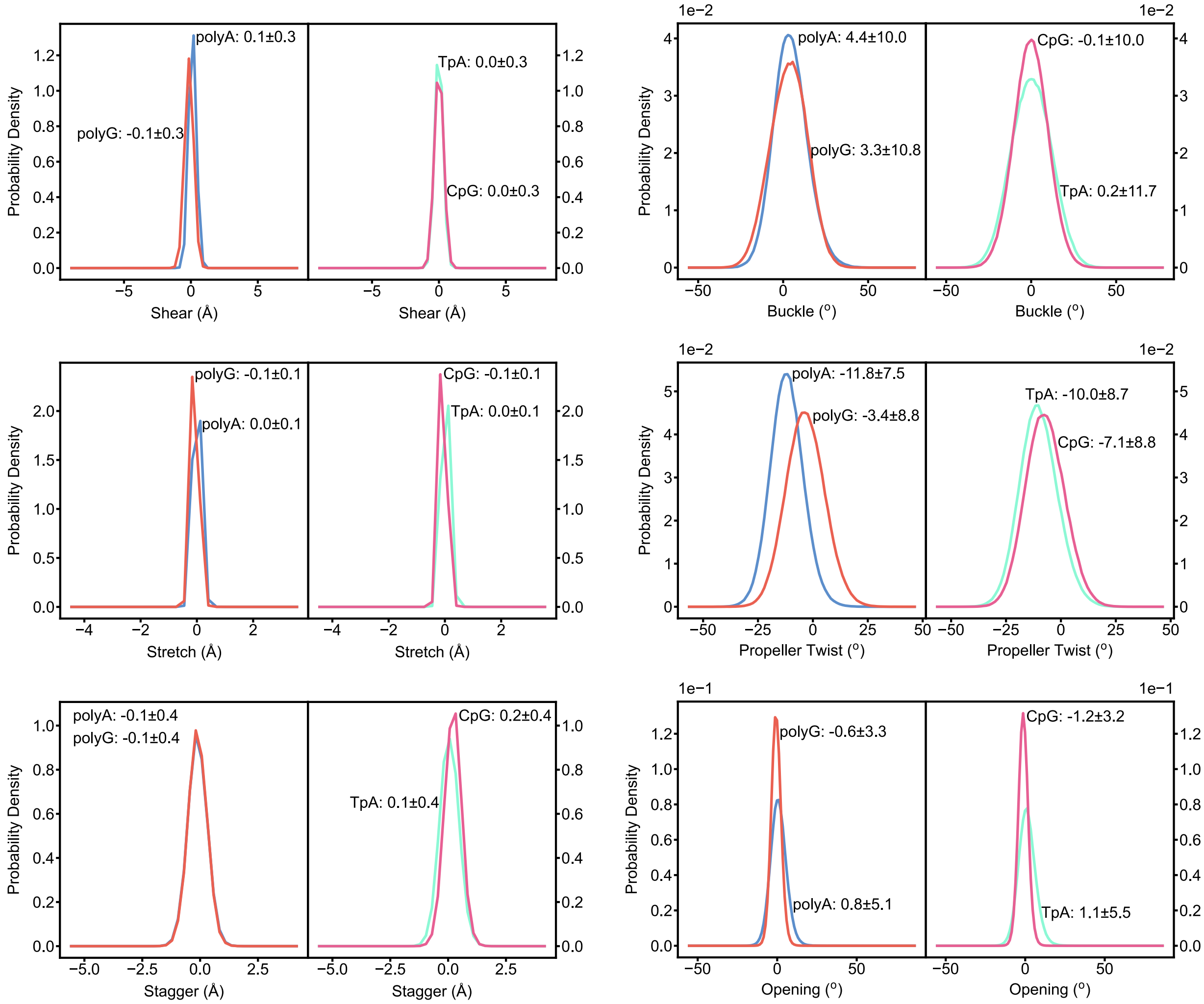

Figure S17: The characterization of base pair parameters in the MD simulations of the transcription regulatory sequences.

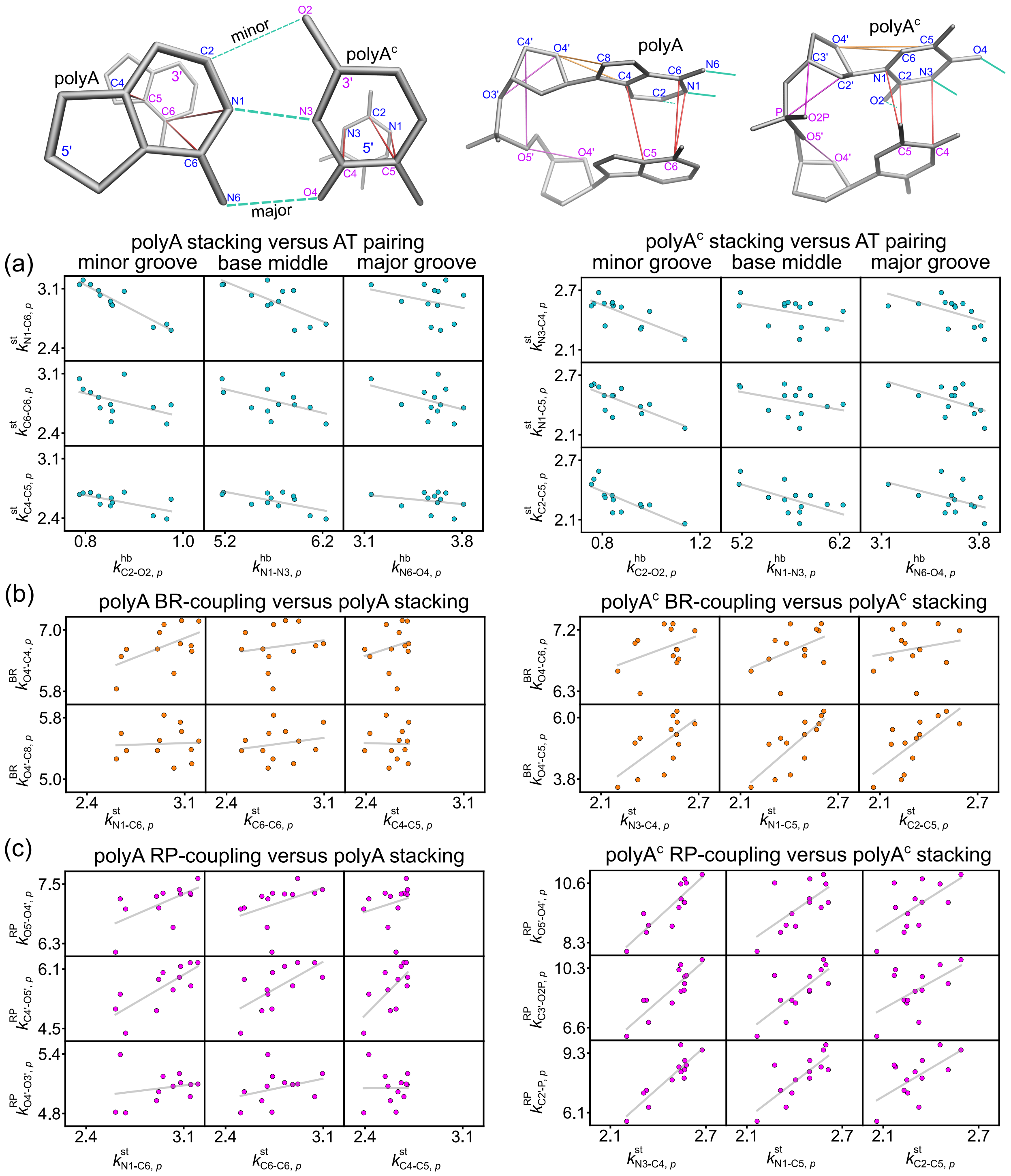

Figure S18: The correlation in coupling strengths (kcal/mol/Å^2) between different mechanical compartments in polyA and polyA^c. The molecular illustrations of $\{$a-b$\}^m$ (m=hb, st, BR and RP) are shown in the top panel. (a) $k^{st}_{\text{a-b},p}$ - $k^{hb}_{\text{a-b},p}$ plots, (b) $k^{BR}_{\text{a-b},p}$ - $k^{st}_{\text{a-b},p}$ plots and (c) $k^{RP}_{\text{a-b},p}$ - $k^{st}_{\text{a-b},p}$ plots. For m=hb and m=BR, $k^{m}_{\text{a-b},p}$ is the average of force constant over all trajectory windows for the atom pair a-b at base p. For m=st and m=RP, $k^{m}_{\text{a-b},p}$ is the average of force constant over all trajectory windows for the atom pair a-b whose atom a is at base p and atom b is at base $p+1$.

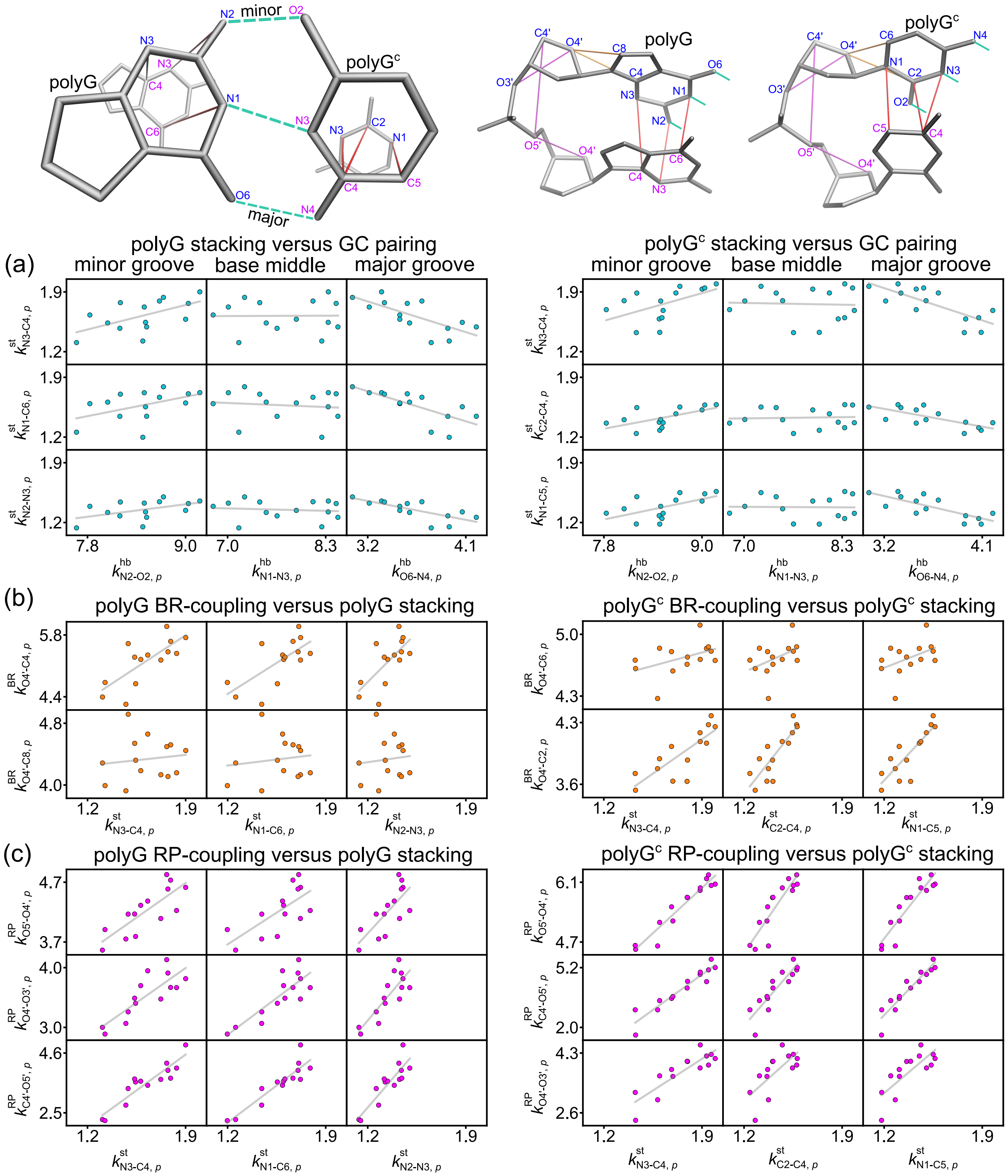

Figure S19: The correlation in coupling strengths (kcal/mol/Å^2) between different mechanical compartments in polyG and polyG^c. The molecular illustrations of $\{\text{a-b}\}^{\text{m}}$ (m=hb, st, BR and RP) are shown in the top panel. (a) $k^{\text{st}}_{\text{a-b},p}$ - $k^{\text{hb}}_{\text{a-b},p}$ plots, (b) $k^{\text{BR}}_{\text{a-b},p}$ - $k^{\text{st}}_{\text{a-b},p}$ plots and (c) $k^{\text{RP}}_{\text{a-b},p}$ - $k^{\text{st}}_{\text{a-b},p}$ plots. For m=hb and m=BR, $k^{\text{m}}_{\text{a-b},p}$ is the average of force constant over all trajectory windows for the atom pair a-b at base p. For m=st and m=RP, $k^{\text{m}}_{\text{a-b},p}$ is the average of force constant over all trajectory windows for the atom pair a-b whose atom a is at base p and atom b is at base $p+1$.

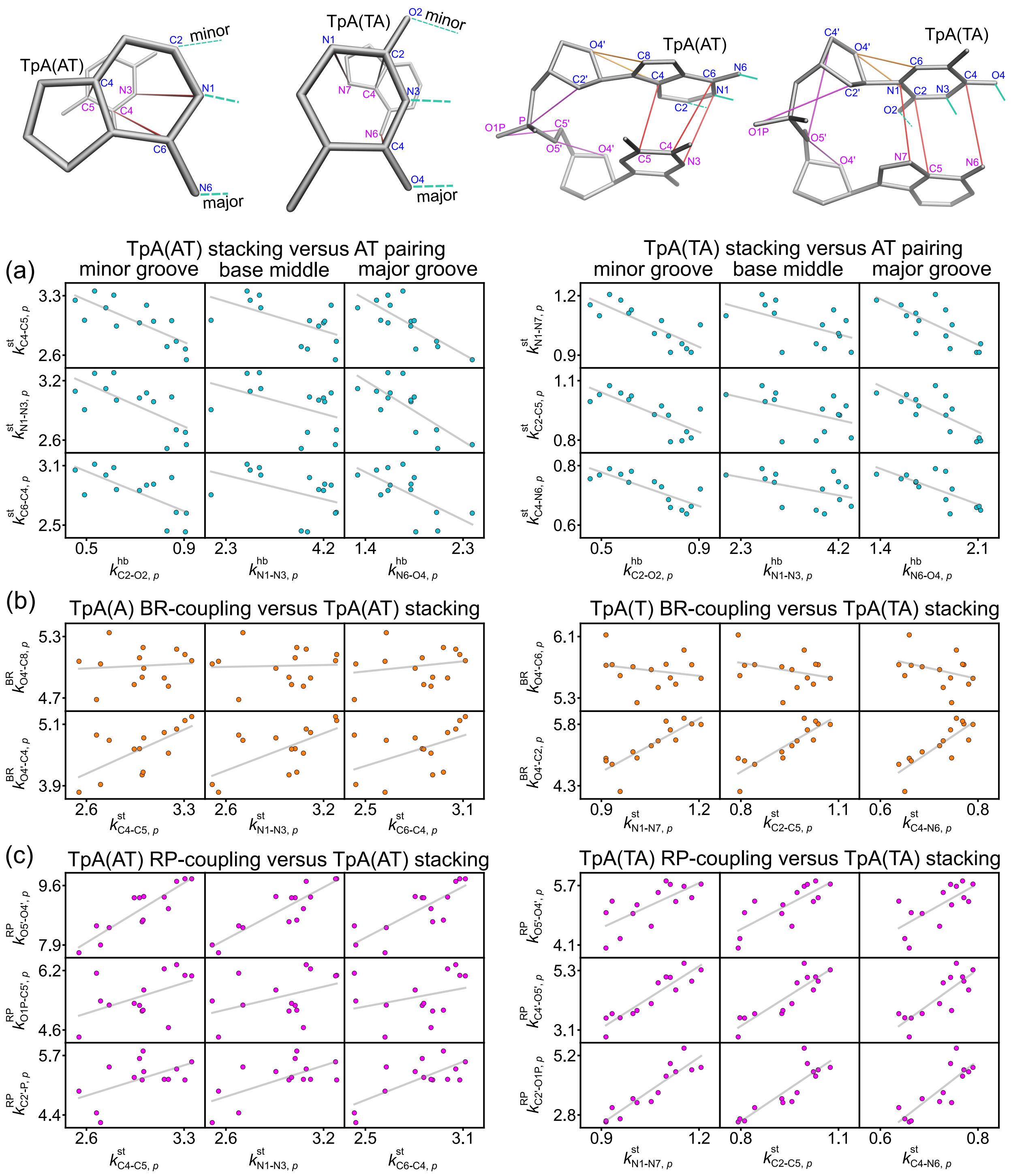

Figure S20: The correlation in coupling strengths (kcal/mol/Å^2) between different mechanical compartments in TpA(AT) and TpA(TA). The molecular illustrations of $\{\text{a-b}\}^{\text{m}}$ (m=hb, st, BR and RP) are shown in the top panel. (a) $k^{\text{st}}_{\text{a-b},p}$ - $k^{\text{hb}}_{\text{a-b},p}$ plots, (b) $k^{\text{BR}}_{\text{a-b},p}$ - $k^{\text{st}}_{\text{a-b},p}$ plots and (c) $k^{\text{RP}}_{\text{a-b},p}$ - $k^{\text{st}}_{\text{a-b},p}$ plots. For m=hb and m=BR, $k^{\text{m}}_{\text{a-b},p}$ is the average of force constant over all trajectory windows for the atom pair a-b at base p. For m=st and m=RP, $k^{\text{m}}_{\text{a-b},p}$ is the average of force constant over all trajectory windows for the atom pair a-b whose atom a is at base p and atom b is at base $p + 1$.

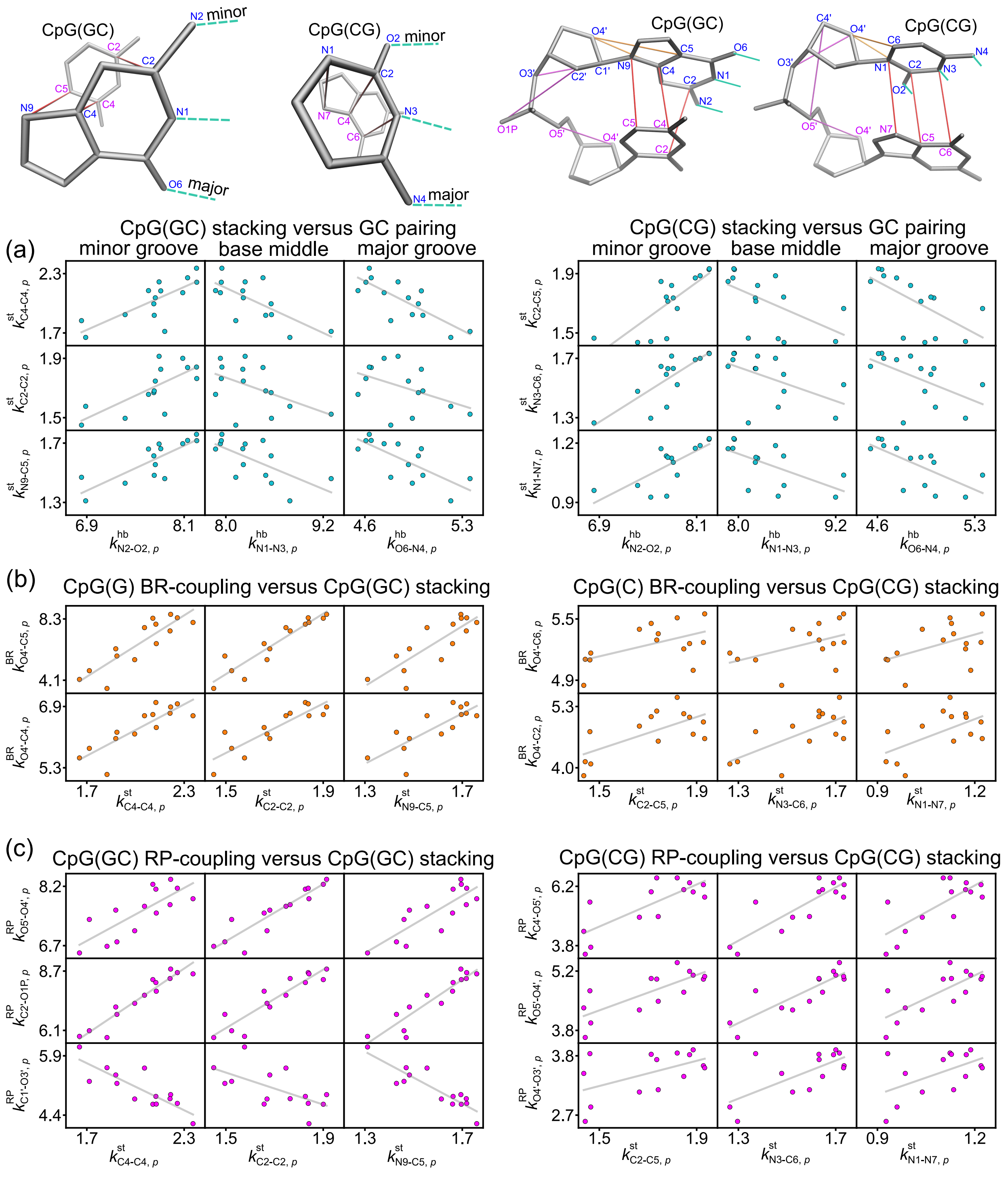

Figure S21: The correlation in coupling strengths (kcal/mol/Å^2) between different mechanical compartments in CpG(GC) and CpG(CG). The molecular illustrations of {a-b}^{m} (m=hb, st, BR and RP) are shown in the top panel. (a) $k^{\mathrm{st}}_{\text{a-b},p}$ - $k^{\mathrm{hb}}_{\text{a-b},p}$ plots, (b) $k^{\mathrm{BR}}_{\text{a-b},p}$ - $k^{\mathrm{st}}_{\text{a-b},p}$ plots and (c) $k^{\mathrm{RP}}_{\text{a-b},p}$ - $k^{\mathrm{st}}_{\text{a-b},p}$ plots. For m=hb and m=BR, $k^{\mathrm{m}}_{\text{a-b},p}$ is the average of force constant over all trajectory windows for the atom pair a-b at base p. For m=st and m=RP, $k^{\mathrm{m}}_{\text{a-b},p}$ is the average of force constant over all trajectory windows for the atom pair a-b whose atom a is at base p and atom b is at base $p+1$.

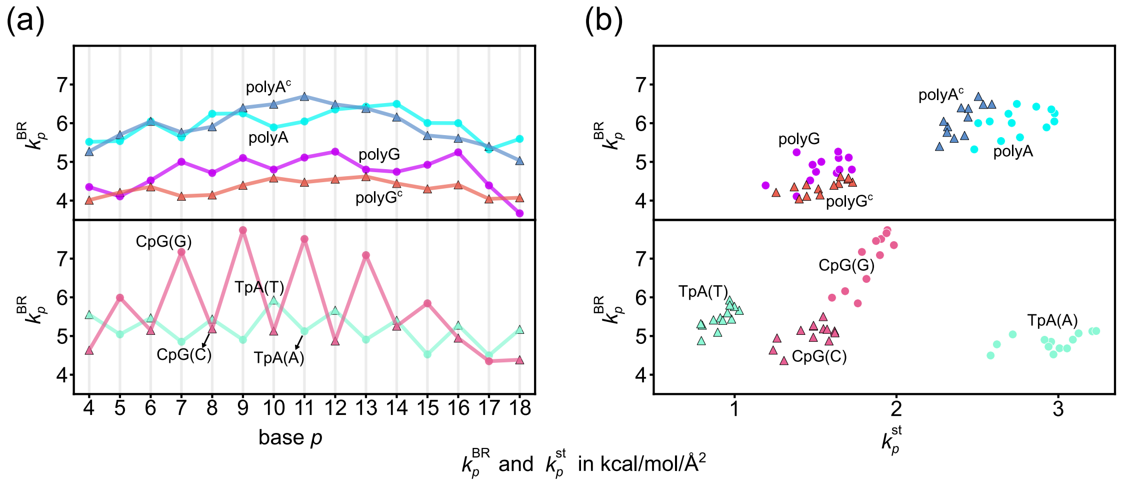

Figure S22: The rigidity of base-ribose linkage (BR) in the transcription regulatory sequences. (a) k_p^{BR} along the sequence index p. The flat profiles of the k_p^{BR}-versus-p curves of polyA and polyG indicate little difference between their homopolymeric strands. The k_p^{BR}-versus-p curves of TpA and CpG sequences, on the other hand, show qualitatively different behaviors. (b) The k_p^{BR}-k_p^{st} plots of polyA, polyA^{c}, polyG and polyG^{c} (top) and of TpA(A), TpA(T), CpG(G) and CpG(C) (bottom). For the polyA bases that have higher stacking rigidity, their k_p^{BR} values are also higher than those in polyG. While TpA stacking exhibits drastic alternation in k_p^{st}, the up-and-down in k_p^{BR} is minor with the TpA(A) rigidity being only slightly lower than the k_p^{BR} values of TpA(T). CpG, on the contrary, has much milder alternation in the stacking rigidity, but the k_p^{BR} up-and-down is significantly more pronounced than that in TpA. Furthermore, the k_p^{BR} values of CpG(G) are evidently higher than those of CpG(C), a trend opposite to that in TpA.

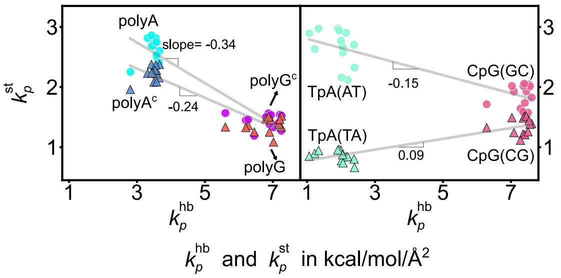

Figure S23: The k_p^{st}-k_p^{hb} plots of polyA, polyA^{c}, polyG, and polyG^{c} (left) and of TpA(AT), TpA(TA), CpG(GC) and CpG(CG) (right). The linear best fit of k_p^{st} to k_p^{hb} is shown for each group of (polyA, polyG), (polyA^{c}, polyG^{c}), (TpA(AT), CpG(GC)), and (TpA(TA), CpG(CG)). The values of k_p^{hb} and k_p^{st} are calculated from 1 μs all-atom MD simulations of all sequences using the OL15 force field. The other details remain the same as in the data processing of Fig. 4b.

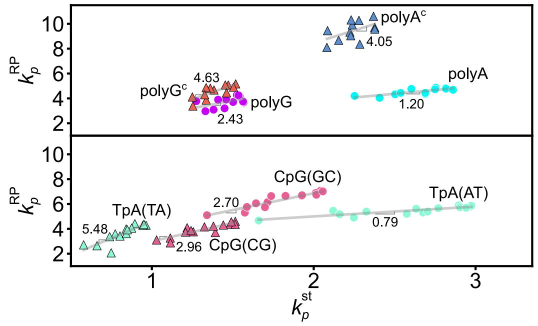

Figure S24: The k_p^{RP}-k_p^{st} plot of polyA, polyA^{c}, polyG, and polyG^{c} (top) and of TpA(AT), TpA(TA), CpG(GC) and CpG(CG) (bottom). The linear best fit of k_p^{RP} to k_p^{st} is shown for each sequence motifs. The values of k_p^{st} and k_p^{RP} are calculated from 1 μs all-atom MD simulations of all sequences using the OL15 force field. The other details remain the same as in the data processing of Fig. 6a (bottom).

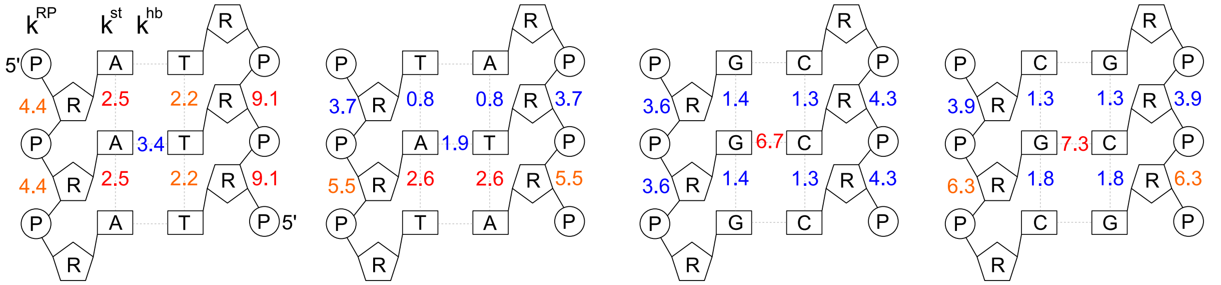

Figure S25: The trigram of base-to-backbone rigidity for polyA, polyG, TpA, and CpG in terms of the k^{hb}, kst and k^{RP} values in kcal/mol/Å^2. All mean inter-moiety rigidities are calculated from the 1 μs all-atom MD simulation of each sequence using the OL15 force field. The other details remain the same as in the data processing of Fig. 7.

References

(S1) Lavery, R.; Moakher, M.; Maddocks, J. H.; Petkeviciute, D.; Zakrzewska, K. Conformational analysis of nucleic acids revisited: Curves+. *Nucleic Acids Res.* **2009**, *37*, 5917–5929.

(S2) Gittes, F.; Mickey, B.; Nettleton, J.; Howard, J. Flexural rigidity of microtubules and actin filaments measured from thermal fluctuations in shape. *J. Cell Biol.* **1993**, *120*, 923–934.
